# Infrared photoconduction at the diffusion length limit in HgTe nanocrystal arrays

**DOI:** 10.1038/s41467-021-21959-x

**Published:** 2021-03-19

**Authors:** Audrey Chu, Charlie Gréboval, Yoann Prado, Hicham Majjad, Christophe Delerue, Jean-Francois Dayen, Grégory Vincent, Emmanuel Lhuillier

**Affiliations:** 1 Sorbonne Université, CNRS, Institut des NanoSciences de Paris, Paris, France; 2 ONERA - The French Aerospace Lab, Palaiseau, France; 3 Université de Strasbourg, IPCMS-CNRS UMR 7504, Strasbourg, France; 4 Univ. Lille, CNRS, Centrale Lille, Univ. Polytechnique Hauts-de-France, Junia, UMR 8520 - IEMN, Lille, France; 5 Institut Universitaire de France, Paris, France

**Keywords:** Electronic devices, Nanophotonics and plasmonics

## Abstract

Narrow band gap nanocrystals offer an interesting platform for alternative design of low-cost infrared sensors. It has been demonstrated that transport in HgTe nanocrystal arrays occurs between strongly-coupled islands of nanocrystals in which charges are partly delocalized. This, combined with the scaling of the noise with the active volume of the film, make case for device size reduction. Here, with two steps of optical lithography we design a nanotrench which effective channel length corresponds to 5–10 nanocrystals, matching the carrier diffusion length. We demonstrate responsivity as high as 1 kA W^−1^, which is 10^5^ times higher than for conventional µm-scale channel length. In this work the associated specific detectivity exceeds 10^12^ Jones for 2.5 µm peak detection under 1 V at 200 K and 1 kHz, while the time response is as short as 20 µs, making this performance the highest reported for HgTe NC-based extended short-wave infrared detection.

## Abstract (web summary)

Infrared nanocrystals have become an enabling building block for the design of low-cost infrared sensors. Here, Chu et al. design a nanotrench device geometry at the diffusion length limit in HgTe nanocrystals and demonstrate the record high sensing performance operated in the short-wave infrared.

## Introduction

Infrared (IR) detection enables detection and imaging beyond the eye sensitivity (λ > 800 nm). In the near infrared, silicon can be used up to the material band gap (1100 nm). When longer wavelengths are targeted, narrower band gap bulk semiconductors (Ge, InGaAs, InSb, PbS(e), HgCdTe) or heterostructures of semiconductor (quantum wells and type-II superlattices) are used to achieve low energy photon absorption. IR sensing has for long been a niche topic focused on defense and astronomy applications due to prohibitive device costs. Self-driven cars and industrial vision offer possibilities to expand the applications of IR detection toward larger markets, if low-cost sensors become available. Detectors based on epitaxially-grown semiconductors are now mature and high performing technologies. However, this maturity also makes unlikely cost disruption to happen. Current low-cost and room-temperature operating alternatives such as bolometers also present key limitations (low signal-to-noise ratio and slow-time response) making them incompatible for LIDAR (light detection and ranging) sensing used in self-driving vehicles (requiring fast detection). In the quest for an alternative technology, a large effort has been dedicated to IR nanocrystals (NCs)^1^. Progresses on both material growth^2–6^ and device design^7^ transformed HgTe NCs into a versatile platform for IR optoelectronics^8^. Key developments include on-chip integration^9^, coupling to resonators to achieve strongly-absorbing devices^10–14^ and hybridization to read out circuits to design focal plane arrays operating in the short-wave^15,16^ and mid-wave IR^17,18^.

Among possible device geometries (planar photoconductors, phototransistors^19,20^, and photodiodes^21^) to integrate the NC absorbing layer, vertical photovoltaic devices^7,11^ lead to the highest performance, close to commercial sensors one. The enhancement of performance is attributed to the built-in electric field of the diode, which enables a zero-bias operation and thus a reduction of the dark current and its associated noise. Nevertheless, compared to conventional devices in planar geometry, vertical diodes also rely on a thinner structure and a smaller effective volume. Recently, Lan et al. observed that transport in NC arrays occurs between islands of strongly-coupled nanocrystals^22^. Within an island the transport occurs over a delocalized state, while the bottleneck of the charge conduction is the inter-island hopping step. By shrinking the inter-electrode spacing, the vertical diode reduces the number of critical hopping steps between weakly-coupled islands of NCs. In addition, in NC arrays, the main source of noise is a *1/f* noise^23,24^. Hooge’s law gives^25^ an empirical scaling for the 1/*f* noise magnitude where the noise current spectral density *δI* is given by $\delta I^2 = \frac{{\alpha I^2}}{{Nf}}$, with *α* the Hooge’s constant, *I* the current, *N* the total number of involved carriers and *f* the signal frequency. Because the current magnitude scales with the volume of the device, smaller devices should have a lower noise spectral density. Last, reducing the device size favors photoconduction gain^26–29^. In conventional IR sensors the benefits of gain are generally balanced by an increased noise. However, in NC films, since generation-recombination noise is not prevailing, it is possible to take benefit of the gain-enhanced responsivity without generating more noise. Combined altogether, these three effects (better transport, reduced noise, and photoconductive gain) suggest that smaller devices should be targeted to achieve enhanced detection. Those effects are not exclusive to vertical geometry and also apply to planar photoconductive devices, made of nanocrystal arrays contacted in plane by metallic electrodes. As explained before, one key advantage of vertical devices is to allow shorter inter-electrode distance. However, the manufacturing of vertical devices is relatively complex, requiring many patterning steps, especially when considering the risk of a possible physico-chemical degradation of the active layer, resulting in an increased device cost. This makes planar-type photodetectors, an interesting, versatile, and cheap alternative, with simpler back gate control and direct optical access to the active layer. Hence, reducing the inter-electrode spacing in planar devices appears as a key lock for the development of efficient photoconductive devices.

In this work, we design and fabricate devices based on nanotrenches with inter-electrodes spacing matching the carrier diffusion length^22^. Because, NCs aim to be integrated in low-cost devices, we are cautious to make the fabrication process only based on simple optical lithography procedures (i.e., no e-beam lithography). We demonstrate that the small size of the device shows a significant gain (>10^6^), leading to the largest responsivity reported in HgTe NC films operated in the extended short-wave infrared. We demonstrate that specific detectivity D* as high as 10^12^ Jones is achieved (200 K operation under 1 V for signal at 1 kHz) as the device size is shrunk down to 40 nm. We then use a combination of electrostatic, electromagnetic, and Schrödinger equation simulations to reveal the detailed operation of this device.

## Results

### Active material and its integration onto nano-size devices

**Fig. 1:**
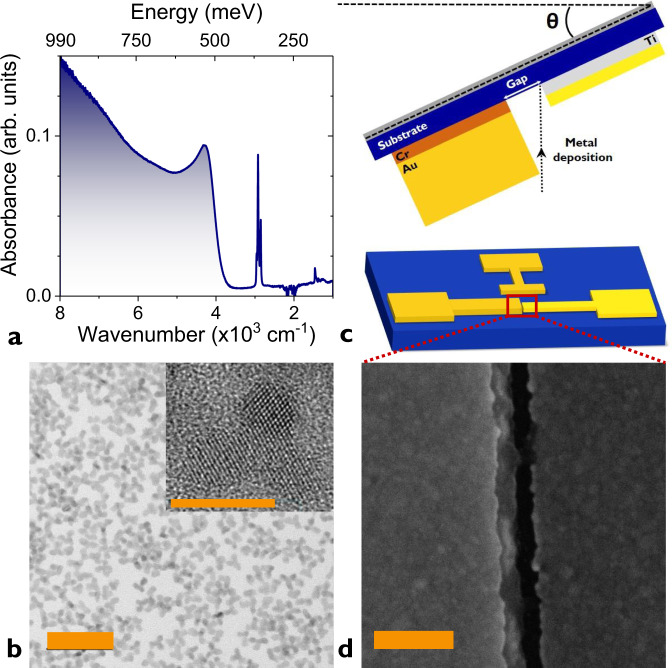
Active material and nanotrench device fabrication. **a** Infrared absorption spectrum of HgTe nanocrystals (NCs) used in this study. The narrow line at 2900 cm^−1^ is due to the C–H bond from the capping ligands. **b** Transmission electron microscopy image of HgTe NCs. The scale bar is 50 nm. The top inset is a high resolution zoom on a HgTe NC. The scale bar is 10 nm. **c** Sketch of the main step of fabrication leading to nanotrench electrodes: tilted evaporation. Bottom sketch is a scheme of the final device. The red rectangle highlight the nanotrench area. **d** Scanning electron microscopy image of 85-nm nanogap in between nanotrench electrodes. The scale bar is 200 nm.

The first step is the growth of HgTe NCs absorbing in the short wave IR. We use the procedure proposed by Keuleyan et al.^2^. This leads to a band-edge around 4000 cm^−1^ or 0.5 eV corresponding to a 2.5-µm cut-off wavelength, see Fig. 1a and Supplementary note 1. The particles are tripods as revealed by transmission electronic microscopy, Fig. 1b. To design electrodes with a very short channel length, we use the procedure developed by Dayen et al.^30,31^ and inspired from single electron transistor fabrication. The methods present two key advantages. First, it only relies on optical lithography despite the sub-wavelength resolution of the final device. Secondly, it enables a high aspect ratio of the channel which is typically difficult to achieve with e-beam methods. The process is depicted in experimental methods and Supplementary methods. The key step to achieve sub-wavelength resolution is a tilted evaporation, see Fig. 1c. After the first lithography, the second electrode is designed to overlap with the first one. To achieve sub-100-nm resolution, this second evaporation is tilted. The interelectrode spacing is the result of a shadowing effect by the first electrode during the evaporation. The first electrode thickness and the tilt angle *θ* set the final nanogap size. Here, we target nanotrenches with nanogap sizes in the 40–85 nm range (see Fig. 1d and Supplementary methods), while their width is as large as few tens of µm. The electrodes are then functionalized by the deposition of an ink^21^ (HgTe NCs capped with short ligands in a polar solvent) to form a 300-nm-thick film covering the nanotrench (see Supplementary note 1).

### Electrical and IR detection properties of nanotrench devices

**Fig. 2:**
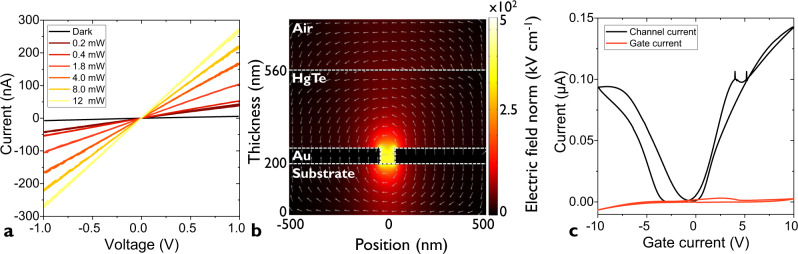
Electrical characterization of the nanotrench electrodes. **a** I–V curves for a nanogap functionalized by a HgTe nanocrystal array in the dark and under illumination by a 1.55 µm laser diode. The experiment is conducted at 200 K. **b** Simulated electric field color map for a 40-nm nanotrench under a 3 V bias. Arrows represent the direction of the electric field. **c** Transfer curves (channel and gate current as a function of applied gate bias) for an HgTe nanocrystal array deposited on a nanotrench fabricated on a LaF_3_ substrate used as a back gate. The measurement is conducted at 200 K.

A first critical step for the use of IR sensors based on nanotrenches, is to confirm that transport is indeed occurring within the nanogap. Even though we obtain a high success ratio, it is critical to ensure that electrodes are not shorted. To do so, a first electrical test is performed without nanocrystal arrays, and demonstrate open-circuit current below the instrumental noise level ≈10 pA. Then, we probe the transport in dark condition and under illumination. The I–V curves appear to be linear, see Fig. 2a (and Supplementary note 4), with a clear positive photoresponse (i.e., an increase of the conductance under illumination). This observation allows us to exclude electrically shorted electrodes. Then an important point is to determine the effective electrical volume of the junction. Electrostatic simulations confirm that the electric field resulting from the source-drain bias application is predominantly applied within the nanogap. In the film above the trench and above the electrodes, the electric field magnitude is significantly reduced, see Fig. 2b: 50 nm above the nanogap, the electric field magnitude is already one order of magnitude lower compared to its maximum value within the trench. Experimental confirmation that transport is essentially driven by the nanogap area rather than by the film above the metallic electrodes is obtained by designing a field effect transistor (FET), see Supplementary methods. In this case, we use a LaF_3_ substrate^32^ which is an ionic glass^33,34^ and has been proven as efficient way to design high capacitance FETs^20,35,36^. We observe a clear current modulation, see Fig. 2c, with an on/off ratio reaching 1.3 × 10^2^. Since the back-gate induced electric field is fully screened on top of the metallic electrodes, the fact that we observe a gate effect further confirms that the nanoparticle array located within the nanogap is the active material of this device.

**Fig. 3:**
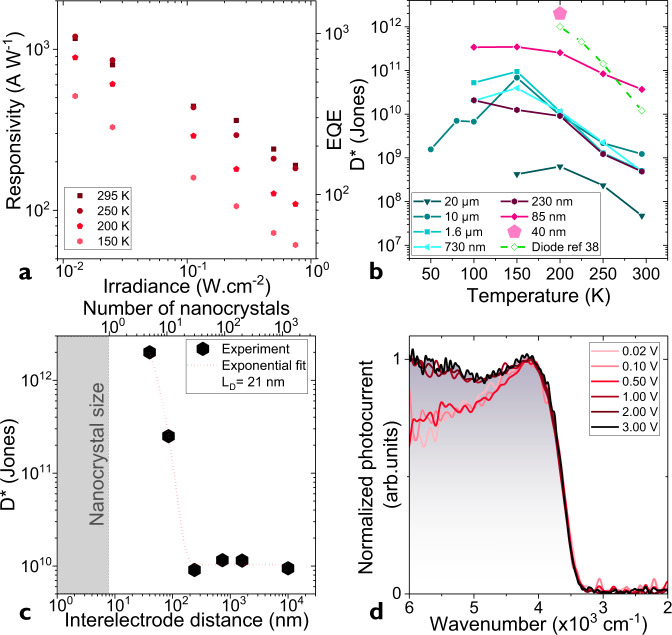
Infrared detection using nanotrench device. **a** Responsivity and external quantum efficiency (EQE) as a function of the incident power for an array of HgTe nanocrystals (NCs) deposited on nanotrench electrodes (nanotrench spacing: 40 nm) at various temperatures. Applied bias is 1 V, while the illumination is ensured by a 1.55 µm laser diode and modulated at 1 kHz. **b** Specific detectivity, D* under 1 V as a function of the temperature for various devices: 10 and 20 µm-spaced interdigitated electrodes, e-beam fabricated electrodes with 230, 730, and 1600 nm spacing and nanotrenches electrodes with 40 and 85 nm spacing. For the sake of comparison with state of the art diode we also provide the performance of the photodiode from ref. ^38^. **c** Specific detectivity measured at 200 K as a function of the electrode spacing. An exponential fit which characteristic decay length is 21 nm is also plotted in red. **d** Photocurrent spectra for an array of HgTe NCs deposited on nanotrench electrodes (electrode spacing: 85 nm) and various applied bias. Measurement is conducted at 200 K.

Now that we have established that the transport of the nanotrench device is driven by the material within the nanotrench, we can test the potential of this device for IR photo-sensing. The responsivity of the device is extremely large, reaching values as large as 1.2 kA W^−1^ under low incident power at room temperature, see Fig. 3a and Supplementary note 4 and 5. Since the illumination is monochromatic (λ = 1.55 µm), we can convert this responsivity into external quantum efficiency (EQE) through the simple expression $R = \frac{{{\mathrm{EQE}} \cdot e}}{{hv}}$, with *e* the proton charge and *hv* the incident photon energy. At this wavelength, 1.2 kA W^−1^ corresponds to an EQE of ≈1000. Since the absorption of the nanogap area has been estimated to be around 0.03% of the incident light from rigorous coupled-wave analysis (RCWA) simulations^37^ (see Supplementary note 7), this corresponds to a gain $g = \frac{{{\mathrm{EQE}}}}{{{\mathrm{abs}}}} \approx 4x10^6$. The gain is also given by the ratio of the minority carrier lifetime by the transit time (i.e., time for a carrier to be conducted from one electrode to the other). The large gain is enabled by the short channel length, which dramatically reduces the transit time.

For the sake of comparison the same film of NCs (same material, same size, same surface chemistry, same operation conditions) deposited on 10 µm-spaced interdigitated electrodes, only presents a responsivity of 1–10 mA W^−1^, see Supplementary note 3. The increase of responsivity (factor ≈ 10^5–6^) resulting from the channel size reduction is much larger than the change of transit time (factor ≈ 500), suggesting that the transport mechanism in such short channel device is impacted. This observation matches the transport mechanism proposed by Lan et al.^22^ who suggests a delocalized transport at short distance, which switches to hopping at longer distances. To better support this change in the transport mechanism, we have probed the specific detectivity of a film of HgTe NCs connected to electrodes with various spacing. In addition to the nanotrench and interdigitated 10-µm-spaced electrodes, we have fabricated electrode with intermediate spacing (240 nm to 1.6 µm) using e-beam lithography (see Supplementary methods and Supplementary note 6). In all devices the noise is found to be *1/f* limited (see Supplementary note 3, 4, and 6). The noise is then used to compare the specific detectivity (i.e., signal-to-noise ratio) for this series of device, see Fig. 3b. Nanotrench devices present an enhancement of the D* by 1 to 2 orders of magnitude and now reach 3 × 10^11^ Jones for a 85 nm-long (size of ≈10 NCs) device and even 2 × 10^12^ Jones for 40 nm-long (size of ≈ 5 NCs) nanogap at 200 K, see Fig. 3c. By fitting the specific detectivity as a function of the electrode spacing using an exponential law, we can extract a characteristic decay length of 21 nm, which corresponds 2–4 nanocrystals depending on their orientation. Such delocalization length confirms that the size of the shortest device matches the characteristic length over which photoconduction occurs.

The obtained specific detectivity value competes with the best HgTe NC-based diode structure^38^ operated in the short-wave IR, see Fig. 3b. The performance can be compared with the one obtained from nanogap devices using lead chalcogenides nanocrystals^28^. Responsivity reaching kA W^−1^ and specific detectivity of 10^12^ Jones has been reported by Lam et al.^29^, but their band gap was twice larger, in this sense our device expand the reachable spectral range at constant performance. This D* value also matches the performance obtained from IR detectors based on epitaxially grow semiconductors while the responsivity is higher, this suggests that the noise magnitude is also higher. This is consistent with the fact that shorter channel device not only magnify the photocurrent but also to a lower extent the dark current. Regarding the operating temperature, we observe an increase of the signal to noise ratio as the device is cooled from 300 K to 150–200 K. Below this temperature range, the specific detectivity seems to plateau and even drops. This behavior relates to the thermal activation of the mobility, which dramatically drops (see Supplementary note 2) at low temperature and is responsible for the observed reduction of the responsivity.

## Discussion

Such high performance requires a better understanding of the device operation. Beyond improved transport and noise reduction, which were the targeted assets of this device, several fundamental additional mechanisms (such as enhanced charge dissociation or electromagnetic field enhancement) may play a role in the nanotrench device operation. First, the small device size induces a large electric field within the nanotrench and may favor the charge dissociation at the NC level.

**Fig. 4:**
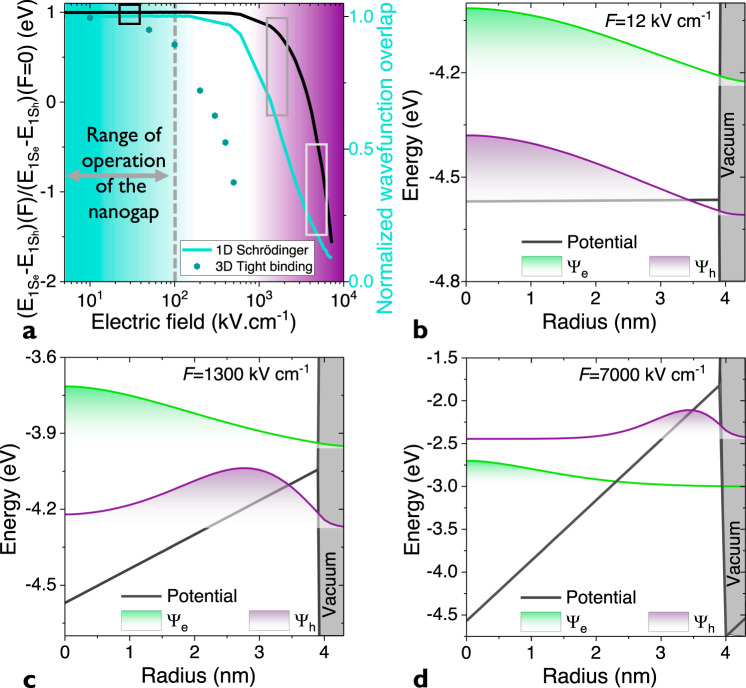
Effect of the electric field on spectra and wavefunction overlaps. **a** Relative change of the band-edge energy (black) and electron-hole overlap (blue) as a function of the applied electric field. **b** (resp **c**, **d**) Energy profile (black) as a well as electron (ψ_e_, blue) and hole (ψ_h_, red) wavefunctions under an electric field (*F*) of 12 kV cm^−1^, (resp 1300 kV cm^−1^ and 7000 kV cm^−1^). The vacuum energy is set to 0 eV. The wavefunctions are shifted to their eigen energy.

The electrodes are operated in a range of electric field that corresponds to a maximum energy drop per NC that coincides with half the HgTe NC optical bandgap (1/2 × 0.5 eV). For this range of bias, we measure the photocurrent spectrum, see Fig. 3d. In particular, we do not observe any shift of the band-edge energy with the applied bias, as it could have been anticipated due to Stark effect. To understand this lack of shift, we solve the Schrödinger equation in the presence of an electric field (*F*) and follow the band gap value (Fig. 4a) as well as the wavefunction localization (Fig. 4b-d). At low fields (*F* = 12 kV cm^−1^, see Fig. 4b), the field-induced energy drop (*e*.*F*.*R*, with *e* the electron charge, *F* the electric field and *R* the particle radius) is small compared to the bandgap *E*_G_ and no localization effect is observed. Under moderate electric fields (*F* = 1300 kV cm^−1^, see Fig. 4c), $e.F.R \simeq E_{\mathrm{G}}$ and we see the hole shifting away from the electron. Under very large electric fields (*F* *=* 7000 kV cm^−1^, see Fig. 4d), the energy of the hole state is now above the one of the electron. This corresponds to a breakdown effect in diodes. From Fig. 4a, we clearly see that for the targeted range of biases (<3 V or ≈500 kV cm^−1^), the bandgap value is not affected at all. This explains the absence of Stark shift in the photocurrent spectrum. Figure 4a also provides the electron-hole wavefunction overlaps and we see that for the same range of electric fields, the wavefunction overlap is only marginally affected. Note that 3D tight-binding simulations (see Supplementary note 8 for more information about the simulations) confirm that up to 100 kV cm^−1^ the change of overlap due to electric field is weak. Thus, we can exclude that the enhancement of the performance of the nanotrench results from an enhanced charge dissociation. Much higher electric fields would be required to observe such phenomenon.

**Fig. 5:**
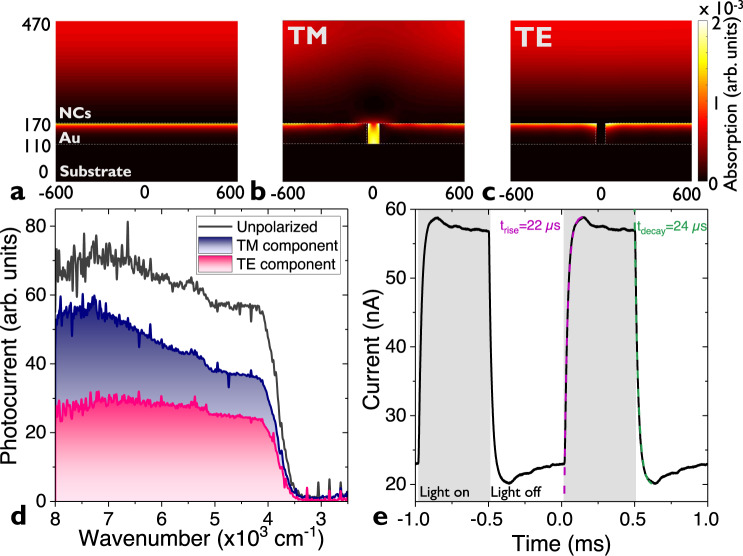
Optical properties of the array of HgTe nanocrystals (NCs) deposited on a nanotrench electrode. **a** Absorption map at 2.5 µm of a 300-nm film of NCs on a gold mirror. **b**. (resp. **c**) Absorption map at 2.5 µm of a 300-nm film of NCs on a 40-nm nanotrench in TM (resp TE) polarization. TM (resp. TE) polarization correspond to the magnetic (resp. electric) field along the trench. The axes are in nm. **d** Photocurrent spectrum for a HgTe NC array deposited on a 40-nm nanotrench electrode with and without a polarizer. The bias is 1 V and measurement is conducted at room temperature. **e** Current as a function of time for a HgTe NC array deposited on a 40-nm nanotrench electrode as the illumination is turned on and off. Pink and green dashed lines correspond to an exponential fit of the rise and the decay as the light is turned on and off. Measured rise and decay time are 22 and 24 µs, respectively. The illumination is ensured by a 1.55 µm laser diode delivering 2 mW of optical power and modulated at 1 kHz. The bias is 1 V.

Another possible mechanism that may play a role in this nanotrench is light focusing. Other shapes of nanotrenches have previously shown electromagnetic field enhancements as large as 10^9 ^^39^. Using RCWA simulations^37^, we first check that the combination of a gold mirror coated by the film of NCs does not lead to Fabry–Perot cavity formation. Indeed, no resonance is observed on the spectrum (see Supplementary note 7) nor in the absorption map, see Fig. 5a. We then introduce the nanotrench in the bottom mirror and now observe a clear enhancement of the light absorption in the transverse magnetic (TM) polarization within the nanotrench (Fig. 5b), while no additional absorption is observed along the transverse electric (TE) polarization (Fig. 5c). The power (i.e., square of the electric field) enhancement with respect to the incident field, in the TM mode is nevertheless modest, with a factor about 10, see Supplementary note 7.

The simulated absorption spectra of the different parts of the device (metallic electrodes, nanotrench and the film on top, see Supplementary note 7) give two insights: (i) the absorption of the nanotrench accounts for only 0.1% (at1.55 µm) of the total absorption of the device and (ii) the polarization effect only comes from the nanotrench. This polarized absorption is confirmed experimentally, by measuring photocurrent spectra along both directions, see Fig. 5d. The fact that we observe a higher contribution from the TM mode further confirms that the active volume of the device comes from the material within the nanotrench. We can conclude that light focusing plays a role in the responsivity enhancement but its contribution is only responsible for a factor 5 (a factor 10 along only one polarization). Last, the time response of the nanogap device is fast, see Fig. 5e. Measured rise and decay times appear to be ≈20 µs, fully consistent with previous report relative to HgTe NC-based detectors found to have a fast responsivity^40^.

To summarize, we design a photoconductive device using HgTe NCs as absorbing material. By reducing the spacing size of the electrodes below 100 nm, and preserving at the same time a large aspect ratio thanks to nanotrench architecture, we observe an increase of the photoresponse by a factor 10^5^. The specific detectivity of the device can reach 10^12^ Jones for low bias operation (1 V) at 200 K, while the time response of the device can be as short as 20 µs. Using FET measurements and electrostatic simulations, we confirm that active area of the device is only localized within the nanogap. Solving Schrödinger equation and using tight-binding simulations, we can exclude field-induced charge dissociation as a possible mechanism involved in the photoresponse enhancement. On the other hand, light focusing leads to a small increase of the light absorption and to a polarized photoresponse. By scaling the size of the device down to the carrier diffusion length, found to be around 20 nm for the surface chemistry used here, we not only generate gain but also reduce the number of hopping steps between weakly connected NC islands leading to the observed enhancement of the response. This work shows that the design of IR sensors should not be limited to diode structures and that simple photoconductive devices can also lead to extremely competitive performance. Future work should focus on the increase of the absorbing area either by extending the nanotrench aspect ratio or by identifying a strategy to collect the light over a much larger area than the nanotrench itself using lens effect for instance.

## Methods

### Chemicals

Mercury chloride (HgCl_2_, Strem Chemicals, 99%), Mercury compounds are highly toxic. Handle them with special care. Tellurium powder (Te, Sigma-Aldrich, 99.99%), trioctylphosphine (TOP, Alfa, 90%), oleylamine (OLA, Acros, 80–90%), dodecanethiol (DDT, Sigma-Aldrich, 98%), methanol (VWR, 98.5%) acetone (VWR rectapur), ethanol (absolute VWR), toluene (VWR, 99.8%), hexane (VWR), dimethylformamide (DMF, VWR, 99.9%), mercaptoethanol (MpOH, Merck, >99%). All chemicals are used without any further purification.

### 1M TOP:Te precursor

2.54 g of Te powder is mixed in 20 mL of TOP in a three-neck flask. The flask is kept under vacuum at room temperature for 5 min and then the temperature is raised to 100 °C. Furthermore, degassing of flask is conducted for the next 20 min. The atmosphere is switched to nitrogen and the temperature is raised to 275 °C. The solution is stirred until a clear orange coloration is obtained. The flask is cooled down to room temperature and the color switches to yellow. Finally, this solution is transferred to an N_2_ filled glove box for storage.

### HgTe NCs synthesis with band edge at 4000 cm^−1^

513 mg of HgCl_2_ was added to 50 mL of oleylamine in a 100 mL three-neck flask. The solution was degassed under vacuum for 1 h at 110 °C. Then, the temperature is decreased to 80 °C and solution is placed to nitrogen atmosphere. 1.9 mL of TOP:Te (1 M) with 10 mL of oleylamine is added to the mercury solution. The solution color gradually turns to dark brown and the reaction is stopped at 3 min. A solution made of 1 mL of dodecanethiol and 9 mL of toluene is quickly added to quench the reaction. The nanocrystals are then precipitated with methanol. After centrifugation, the nanocrystals are redispersed in toluene. The washing step is repeated two more times with ethanol. The solution is filtered with a 0.2 µm filter and redispersed in toluene to reach a 50 mg.mL^−1^ concentration.

### HgTe ink and thin film preparation

15 mg of HgCl_2_, 1 mL of MpOH and 9 mL of DMF are mixed. At 1 mL of this solution is added 6 mL of hexane and 500 µL of a HgTe solution (at 50 mg mL^−1^ in toluene). After stirring the QDs migrate from the top phase (hexane) to the bottom DMF phase, showing an efficient ligand exchange. After 3 washing steps with hexane, few drops of EtOH are added and the solution is centrifuged at 4427 × *g* for 3 min. The supernatant is discarded and the pellet is redispersed in 130 µL of DMF. 35 µL of this solution is used to prepare the film by spin-coating. The nanotrench electrodes are plasma-cleaned for 5 min. Then 35 µL of the ink are deposited on the substrate. The spin-coating steps are 1200 rpm (acceleration 200 rpm s^−1^) for 180 s and 3000 rpm (200 rpm s^−1^) for 120 s. The obtained film thickness is about 300 nm.

### Electron microscopy

For transmission electron microscopy (TEM) pictures, a drop of the CQD solution is drop-casted onto a copper grid covered with an amorphous carbon film. The grid is degassed overnight to reduce future contamination. A JEOL 2010F is used for acquisition of pictures and operated at 200 kV.

For scanning electron microscopy (SEM) pictures, a Zeiss Supra 40 scanning electron microscope is used. The acceleration bias is set at 7 kV and the aperture at 15 µm.

### Infrared spectroscopy

Infrared spectroscopy is conducted using a Fisher IS50 Fourier transform Infrared spectrometer (FTIR). To measure NC absorption, we use the spectrometer in ATR configuration. A drop of NC solution is dried on the diamond cell. The source is a globar, the beamsplitter is an extended-KBr and the detector is a DTGS ATR. Spectra are typically acquired between 8000 cm^−1^ and 400 cm^−1^ with a 4 cm^−1^ resolution and averaging over 32 spectra. Photocurrent spectra are acquired as the sample is biased using a Femto DLPCA 200 current amplifier which role is also to magnify the current. The signal is then fed into the FTIR acquisition board. Spectra are typically acquired between 8000 cm^−1^ and 2000 cm^−1^ with a 4 cm^−1^ resolution and averaging over 64 spectra.

### Nanotrench fabrication

The nanogap electrodes are made using the procedure described by Dayen et al.^30^. The electrodes are prepared using a two-steps lithography procedure. Starting from a cleaned Si/SiO_2_ wafer (400 nm of oxide), AZ5214 resist is spin coated and baked for 90 s at 110 °C on a hot plate. The first pattern is then exposed for 2 s using a UV light (Süss microtec MJB4). The wafer is further baked for 2 min at 125 °C. Then a flood exposure is conducted for 40 s. Development is then made using AZ326 developer for 25 s and then rinsed in water. A O_2_ plasma cleaning is then conducted for 5 min. Metal deposition (Plassys MEB550S) is operated by deposing 6 nm of Cr and 54 nm of gold. The lift off is made by dipping the substrate overnight in acetone. Another step of plasma cleaning and acetone washing is generally performed to obtain a better success ratio. For the second electrodes, the pattern needs to overlap with the first electrodes and the nanotrench will be formed at the interface (see Supplementary methods). The second lithography is done as described before. For the second metal deposition, the evaporation is made under a tilted angle, typically from 50° to 65° depending on the expected nanotrench size. It is worth pointing that evaporation has to be as directive as possible and that sample holder does not rotate during the evaporation. The second deposition is conducted while depositing 5 nm of Ti and 50 nm of gold. The lift-off is also conducted by dipping the samples in acetone overnight.

### Nanotrench characterization

#### DC transport

The sample is connected to a Keithley 2634b, which controls the drain bias (*V*_DS_) and measures the associated current (*I*_DS_). This measure is carried out in the dark or under illumination using 1.55 µm laser diode or a blackbody at 980 °C.

#### Transistor measurement

The sample is connected to a Keithley 2634b, which sets the drain source bias (*V*_DS_), controls the gate bias (*V*_GS_) and measures the associated currents (*I*_DS_ and *I*_GS_). A scheme of the device is given is Supplementary methods.

#### Responsivity measurement

The source is a laser diode at 1.55 µm or a blackbody at 980 °C placed at 40 cm of the sample. The laser-diode spot size is 1.6 mm². The flux can be chopped form 1 Hz to 10 kHz. The photocurrent is measured using Zurich Instruments MFLI lock-in amplifier at 1 V bias. When the black body is used, a germanium filter is utilized to suppress the high energy part of the blackbody spectrum. The total power calculated according to the formula: $P = A_{\mathrm{D}}.\pi .{\it{{\mathrm{cos}}}}\left( \beta \right). {\it{{\mathrm{sin}}}}^2\left( \alpha \right).{\int}_{\lambda _{{\it{min}}}}^{\lambda _{{\it{max}}}} {\frac{{hc^2}}{{\lambda ^5}}} .\frac{1}{{e^{hc/\lambda kT}}}d\lambda$ where *α* is the solid angle illuminated, *β* is the angle of the sample (0° corresponds to sample perpendicular to the light illumination), *A*_D_ is the device area, *h* is the Planck constant, *c* the light velocity, *k* is the Boltzmann constant and *T* the temperature. The light is chopped form 1 Hz to 1 kHz. The photocurrent is measured using Zurich Instruments MFLI lock-in amplifier under 1 V of applied bias. The sample is mounted on the cold finger of a close cycle cryostat. As scheme of the set-up is given in Supplementary methods.

#### EQE calculation

The external quantum efficiency (EQE) is the ratio of the generated photoelectron flux over the flux of incident photon. For a monochromatic wavelength it is simply given by: ${\mathrm{EQE}} = R.\frac{{h\nu }}{e}$, with *R* the responsivity and *hv* the photon energy of the incident light and *e* the elementary charge.

#### Noise measurement

Current from the device (at 1 V bias, kept in the dark) is amplified by a Femto DLPCA-200, then fed into a SRS SR780 signal analyzer. The sample is mounted on the cold finger of a close cycle cryostat.

#### Specific detectivity determination

The specific detectivity (in Jones) of the sample is determined using the formula: $D^ \ast = \frac{{R\sqrt A }}{{S_I}}$, where *R* (in *A* W^−1^) is the responsivity, *S*_*I*_ is the noise (*A* Hz^−1/2^*A* the area of the device (cm²).

### Electrostatic simulation

Calculations have been achieved using COMSOL Multiphysics software with AC/DC module and electric currents physics. For quantum dots, electrical conductivity and relative permittivity are set to 5 × 10^−4^ S m^−1^ and 4, respectively. The mesh is made of triangular elements whose maximum size has been refined is the nanotrench area and is set to 1 nm. Outside the nanotrench, maximum element size is 5 nm. These values are more than sufficient to properly describe the electrostatic phenomenon for this geometry.

### Electromagnetic simulation

Calculations have been achieved with Matlab library based on rigorous coupled-wave analysis (RCWA)^37^. The Maxwell equations are solved in each layer and interface conditions are applied to find the final solution of the whole structure. We considered incoming plane wave under normal incidence, either with transverse magnetic or transverse electric polarization, i.e., with the magnetic field or electric field parallel with the slits of the grating *s*, respectively. Grating is supposed to be invariant along *y* direction, and repeated infinitely along *x* direction with a period of 10 µm. Inputs needed for this simulation are the device structure and the optical index of the different materials (see Supplementary note 7).

### Schrödinger equation resolution

To probe the effect of the electric field on the bandgap and wave functions, we solve the 1D time-independent Schrödinger equation in spherical geometry: $\left[ { - \frac{{\hbar ^2}}{{2m \ast }}\left( {\frac{2}{r}\frac{\partial }{{\partial r}} + \frac{{\partial ^2}}{{\partial r^2}}} \right) + V(r)} \right]\psi (r) = E\psi (r)$ Where *ħ* is the reduced Planck constant, m^*^ the effective mass profile, *V*(*r*) the energy band profile, *E* the eigen energy and *ψ*(*r*) the wavefunction. *V*(*r*) here accounts for a constant part, relative to confinement for which we add on top of which a linear contribution that mimic the effect of electric field. Using spherical geometry in presence of an electric field can only be valid in the regime where electric field is a small perturbation (*e.F.R* << *E*_G_), which actually corresponds to the operating bias range of our nanotrench electrodes. At large electric field, quantitative results become inaccurate but the charge localization effect remains valid. The equation is solved using a shooting method^41^. Material input parameters are the effective masses of electrons (m*_HgTe_ = 0.035*m*_*0*_^42,43^*)* and holes (m*_HgTe_ = 0.5*m*_*0*_^42,43^) and the position of the valence band maximum of the two materials.

### Tight binding simulations

We used the tight-binding model of ref. ^44^ to calculate the electronic structure of the HgTe NCs. Each Hg or Te atom is described by a double set of sp^3^d^5^s* orbitals, one for each spin orientation. Surfaces are saturated by pseudo-hydrogen atoms characterized by a single s orbital. Tight-binding parameters, i.e., on-site energies, nearest-neighbor hopping matrix elements and spin-orbit coupling terms were determined to provide a very good description of the band structure of bulk HgTe (at 300 K). For all NCs, we calculated 60 (1200) conduction (valence) states and we computed dipolar matrix elements between them as described in ref. ^44^. More information on the overlap definition are given in Supplementary note 8.

## Supplementary information

Supplementary Information

## Data Availability

The data that support the findings of this study are available upon reasonable request.

## Code availability

The code used for the electromagnetic simulation is a Matlab library in open acces^37^ and calculations have also been done using COMSOL Multiphysics software with RF Module and Electromagnetic Waves, Frequency Domain physics. Electrostatic simulations are conducted using COMSOL Multiphysics software with AC/DC Module and Electric Currents physics. The codes used for the Schrödinger equation resolution and tight binding simulations that support the findings of this study are available upon reasonable request.
