# Supplementary material for: Infrared photoconduction at the diffusion length limit in HgTe nanocrystal arrays
Source: Nat Commun. 2021 Mar 19;12:1794. doi: 10.1038/s41467-021-21959-x (PMC7979921; doi:10.1038/s41467-021-21959-x)
Supplement: Supplementary file 1 — Supplementary Information [file 41467_2021_21959_MOESM1_ESM.pdf]

Infrared Photoconduction at the Diffusion Length Limit in HgTe Nanocrystal Array

Audrey Chu[1,2], Charlie Gréboval[1], Yoann Prado[1], Hicham Majjad[3], Christophe Delerue[4], Jean-Francois Dayen[3,5], Grégory Vincent[2], Emmanuel Lhuillier[1*]

[1] Sorbonne Université, CNRS, Institut des NanoSciences de Paris, 4 place Jussieu, 75005 Paris, France.
[2] ONERA - The French Aerospace Lab, 6, chemin de la Vauve aux Granges, BP 80100, F-91123 Palaiseau, France.
[3] Université de Strasbourg, IPCMS-CNRS UMR 7504, 23 Rue du Loess, 67034 Strasbourg, France
[4] Univ. Lille, CNRS, Centrale Lille, Univ. Polytechnique Hauts-de-France, Junia, UMR 8520 - IEMN, F-59000 Lille, France
[5] Institut Universitaire de France, 1 rue Descartes, 75231 Paris cedex 05, France

*To whom correspondence should be sent: el@insp.upmc.fr

Supplementary Methods
Supplementary Note 1: **HgTe Nanocrystals properties**
Supplementary Note 2: **Mobility of HgTe NC thin film**
Supplementary Note 3: **HgTe film onto interdigitated 10-µm spaced electrodes**
Supplementary Note 4: **HgTe film on nanotrench electrodes**
Supplementary Note 5: **Statistic of nanotrench**
Supplementary Note 6: **Devices with intermediate size**
Supplementary Note 7: **Electromagnetic simulations**
Supplementary Note 8: **Tight binding simulations**
Supplementary references

SUPPLEMENTARY INFORMATION

Supplementary Methods

Nanotrench fabrication

The nanotrench electrodes are made using the procedure described by Dayen *et al*.[1] Scheme of the different steps are given Supplementary figure 1. A first lithography and evaporation steps are conducted to deposit the drain and gate electrodes on the substrate (*Supplementary figure 1*a). The surface of Si/SiO_2 wafer (400 nm oxide layer) is cleaned by sonication in acetone. The wafer is rinsed with acetone, then isopropanol and dried with a N_2 gun. A final cleaning is made using an O_2 plasma. An adhesion promoter (TI PRIME) is spin-coated on the substrate and baked at 120 °C for 2 min. AZ5214E is spin-coated and baked at 110 °C for 90 s. The substrate is exposed under UV through a patterned mask for 1.5 s. The film is then baked at 125 °C for 2 min in order to invert the resist. Then a 40 s flood exposure is performed. The resist is developed using a bath of AZ726 for 32 s, rinsed in pure water and dried with N_2. We then deposit 5 nm of chromium and 80 nm of gold using thermal evaporation. The lift-off is performed by dipping the film in acetone for 1 hour. The evaporation of this first step needs to be as directive as possible, to have sharp edges in order to induce a clean shadowing effect during the second evaporation. During the second lithography step, the source electrode mask needs to overlap with the first electrode, see *Supplementary figure 1*b. This ensures the possibility of shadowing effect during the following tilted evaporation. The size of the nanogap is roughly given by $h \tan\theta$, with h the first electrodes thickness and θ the tilting angle. This procedure allows sub-100-nm resolution while using optical lithography. The lift off is this time conducted overnight in acetone. A scheme of the final device is given *Supplementary figure 1*c. To increase the success ratio, 4 nanotrench electrodes are made on each substrate.

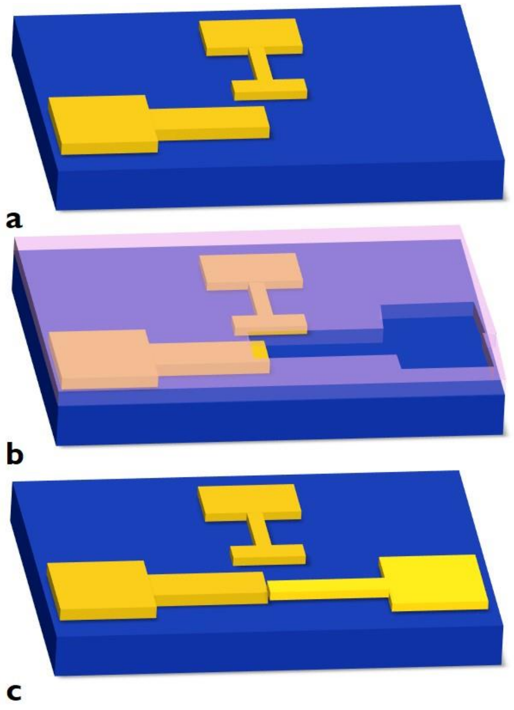

Supplementary figure 1: Scheme of the different steps of fabrication of the nanotrench electrodes. a *Scheme of the nanotrench substrate after the first lithography and evaporation*

steps. ***b*** *Second step of lithography. The pink part is the resist on the substrate.* ***c*** *Scheme of the final device after the tilted evaporation and lift off.*

Two types of nanotrench devices are made. The first ones are made on Si/SiO_2 substrates (see *Supplementary figure 2*a) and the nanotrench length is around 85 nm, see *Supplementary figure 2*b. The second ones are made onto LaF_3 substrate in order to use the substrate as a gate for field effect transistor (FET) measurement, see *Supplementary figure 2*c. The nanotrench length is about 40 nm, see *Supplementary figure 2*d.

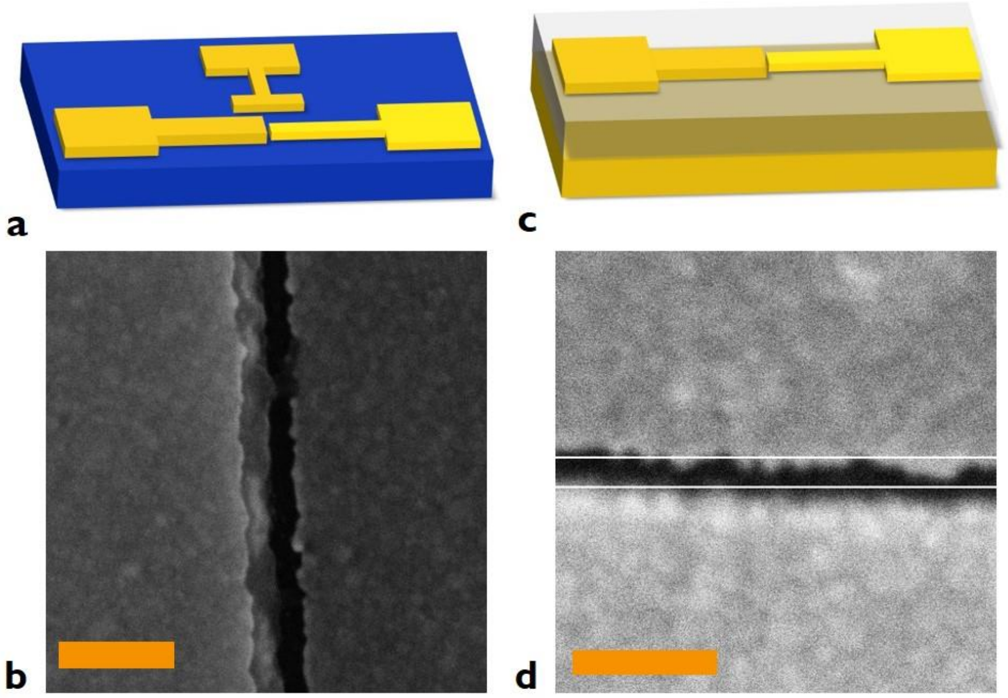

Supplementary figure 2: Presentation of both types of nanotrench devices. a *(resp* ***c****) Scheme of the nanotrench device on* Si/SiO_2 *substrate (resp. onto* LaF_3 *substrate).* ***b*** *(resp* ***d****) SEM image of a 85-nm nanotrench on* Si/SiO_2 *(resp a 40-nm nanotrench on* LaF_3*). The scalebar is 200 nm.*

Interdigitated electrode fabrication

The surface of Si/SiO_2 wafer (400 nm oxide layer) is cleaned by sonication in acetone. The wafer is rinsed with acetone, then isopropanol and dried with a N_2 gun. A final cleaning is made using an O_2 plasma. An adhesion promoter (TI PRIME) is spin-coated on the substrate and baked at 120 °C for 2 min. AZ5214E is spin-coated and baked at 110 °C for 90 s. The substrate is exposed under UV through a patterned mask for 1.5 s. The film is then baked at 125 °C for 2 min in order to invert the resist. Then a 40 s flood exposure is performed. The resist is developed using a bath of AZ726 for 32 s, rinsed in pure water and dried with N_2. We then deposit 5 nm of chromium and 80 nm of gold using thermal evaporation. The lift-off is performed by dipping the film in acetone for 1 hour. The electrodes are rinsed using isopropanol and dried using a N_2 gun. The electrodes are 2.5 mm long spaced by 20 µm. A scheme of this device is given in *Supplementary figure* 3. These electrodes are using for photoconductive devices and electrolyte-gated transistor measurements.

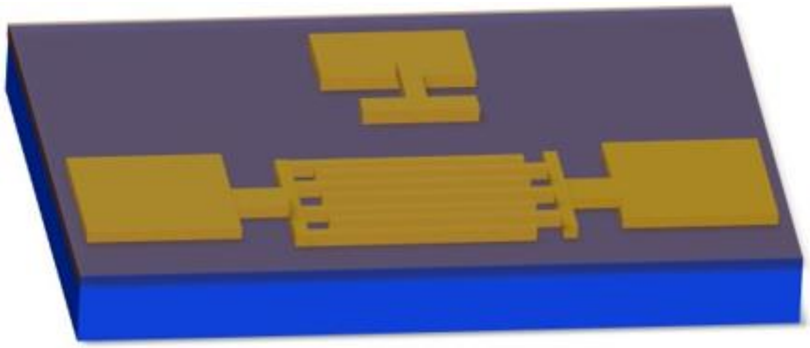

Supplementary figure 3: Scheme of the interdigitated electrode with 10 or 20 µm spacing.

Devices with intermediate size fabrication

To determine the scaling of the transport and phototransport as a function of the inter-electrode spacing, a series of electrodes with spacing between 250 nm and 1.6 µm has been fabricated, see *Supplementary figure 4*. The strategy based on titled evaporation is no longer suitable for such large gaps, while sub µm patterning is not compatible with optical lithography. We thus chose to use e-beam patterning. The fabrication is conducted along a two-step process. First, the main contact pads are fabricated using optical lithography. Then e-beam lithography is used for the electrode with the design of the small trench.

Step 1 Deposition of macroscopic pads for electrical connection. After having rinsed the sample with acetone and isopropanol, the adhesion promoter and the resist are deposited as described in Supplementary Methods 1. The sample is then exposed through a second chromium mask for 1.5 s. The same resist inversion process as described in previous paragraph (annealing + flood exposure) is performed before developing the sample in AZ726 developer for 25 s and rinsing it in pure water. The patterned sample is then put in a VINCI thermal evaporator for a 5 nm Cr and a 80 nm Au depositions. At the end, the sample is dipped in acetone overnight to remove remaining resist. The macroscopic pads for future characterization are made.

Step 2 Fabrication of the electrodes with 250 nm to 2µm spacing. After having rinsed the sample with acetone and isopropanol, a layer of A6 PMMA 950 is spin-coated onto the substrate and baked for 15 min at 150 °C. The samples are transferred in a Zeiss Supra 40 SEM with Raith elphy quantum device for electron beam lithography. The operating bias is set to 20 kV and the aperture to 10 µm. The current is measure at 20 pA. The dose is set at 160 µC cm^{-2}. The PMMA is developed by dipping the film in a solution of MIBK:isopropanol (1:3) for 45 s and rinsed in pure isopropanol for 20 s. We then deposit a 5 nm layer of chromium and a 50 nm layer of gold using thermal evaporation. The lift-off is performed by dipping the film in acetone overnight.

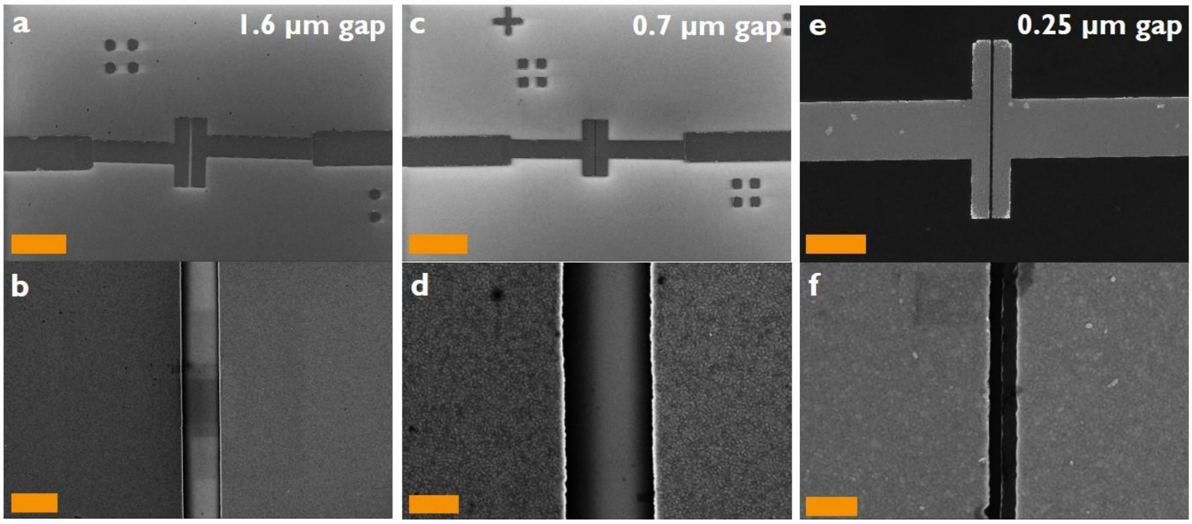

Supplementary figure 4*: *Electron Microscopy image of intermediate size electrode. *Scanning electron microscopy images of 1.6 µm (**a** and **b**), 700 nm spaced (**c** and **d**) and 250 nm (**e** and **f**) electrodes. The scale bars have respectively 30 µm (**a**), 2 µm (**b**), 40 µm (**c**), 400 nm (**d**), 10 µm (**e**) and 400 nm (**f**).*

Electrolyte gating

In a N_2 glovebox, 0.5 g of $LiClO_4$ and 2.3 g of PEG are mixed in a vial. Thin vial is heated at 170 °C on a hot plate for 2 h until the solution becomes clear. To use the electrolyte, the solution is warmed up around 100 °C and brushed on the NC film. A scheme of the electrolyte-gated transistor is given in inset of Supplementary figure 7b.
The sample is connected to a Keithley 2634b, which sets the drain source bias (V_{DS}), controls the gate bias (V_{GS}) with a step of 1 mV and measure the associated currents (I_{DS} and I_{GS}).

Experimental setups

In *Supplementary figure 5* the scheme of a FET using an ionic glass (LaF_3) gate is given. The channel is made of an HgTe thin film. Both electrodes (drain and sources) are connected to a 2634b Keithley sourcemeter. The bias is set at 0.5 V. The substrate made of an ionic glass, LaF_3, is also used as gate. On the back side, a gold plane is deposited and used as gate electrode. This electrode and the source are connected to the other channel of the Keithley sourcemeter.

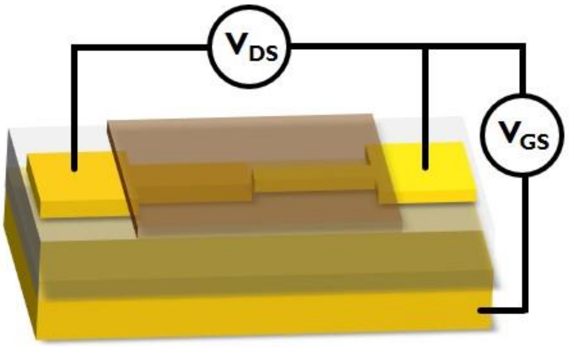

Supplementary figure 5: Scheme of the LaF_3 FET.

In *Supplementary Figure 6* the scheme of the set-up used to measure the responsivity is given. The sample is placed in a cryostat and illuminated with a 1.55 µm laser diode. The illumination is electrically chopped at 1 kHz. Bias is applied on the sample using a sourcemeter. The gate bias can be connected as well. The photocurrent is amplified using a Femto DLPCA-200 transimpedance amplifier and sent to either a scope or a lock-in amplifier. The lock-in (or the scope) is trigged using the signal driving the laser diode.

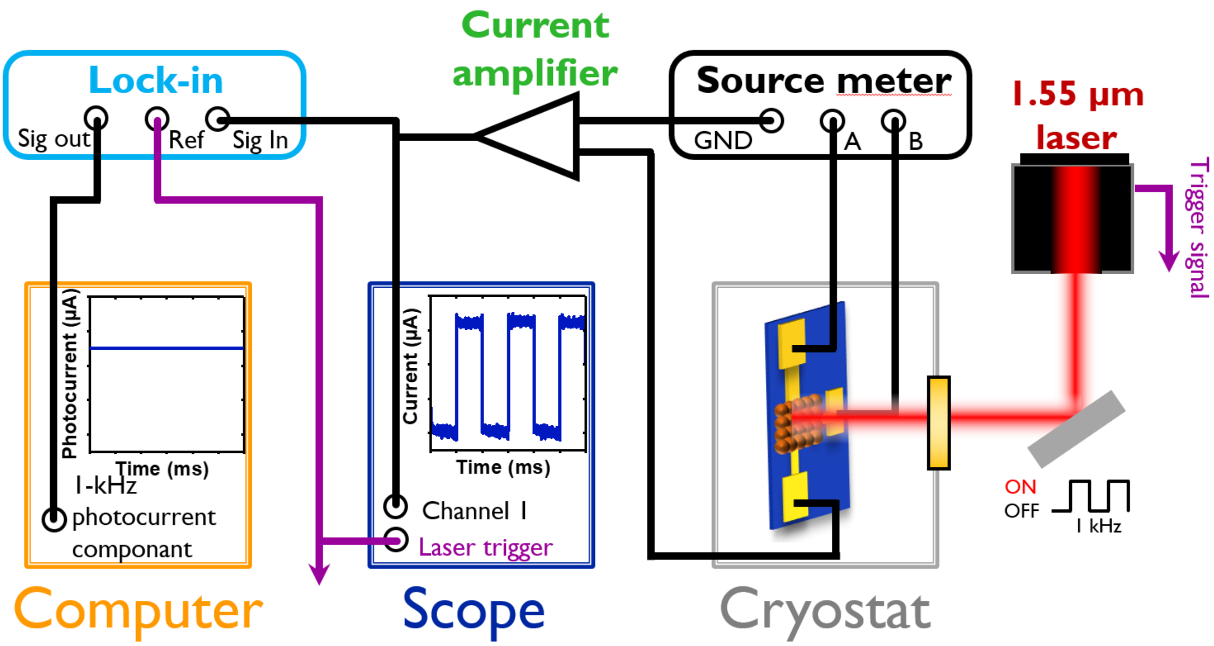

Supplementary Figure 6: Experimental set-up of the responsivity measurement.

Supplementary Note 1: **HgTe Nanocrystals properties**

HgTe nanocrystals (NCs) used in this study have a cut-off at 4000 cm^{-1} (or 2.5 µm or 0.5 eV), see *Supplementary figure 7*. This material can be further studied by photoemission to extract the exact band alignment,[2] see *Supplementary figure 7*f. The fermi level position (with respect of the vacuum level) is found to be -4.57 eV, see *Supplementary figure 7*d. This value can be later input as the potential in the Schrödinger equation for simulations. The energy difference between the valence band and the Fermi level is 0.22 eV, see *Supplementary figure 7*e. On a 300-nm HgTe NC film (see *Supplementary figure 7*c), an electrolyte FET is conducted, see *Supplementary figure 7*b. From the energy diagram and FET measurements, it is clear that HgTe NCs have an ambipolar behavior (*i.e.* conduct electrons and holes), *Supplementary figure 7*b and f. All photoemission measurements were conducted at Tempo beam line at synchrotron SOLEIL.

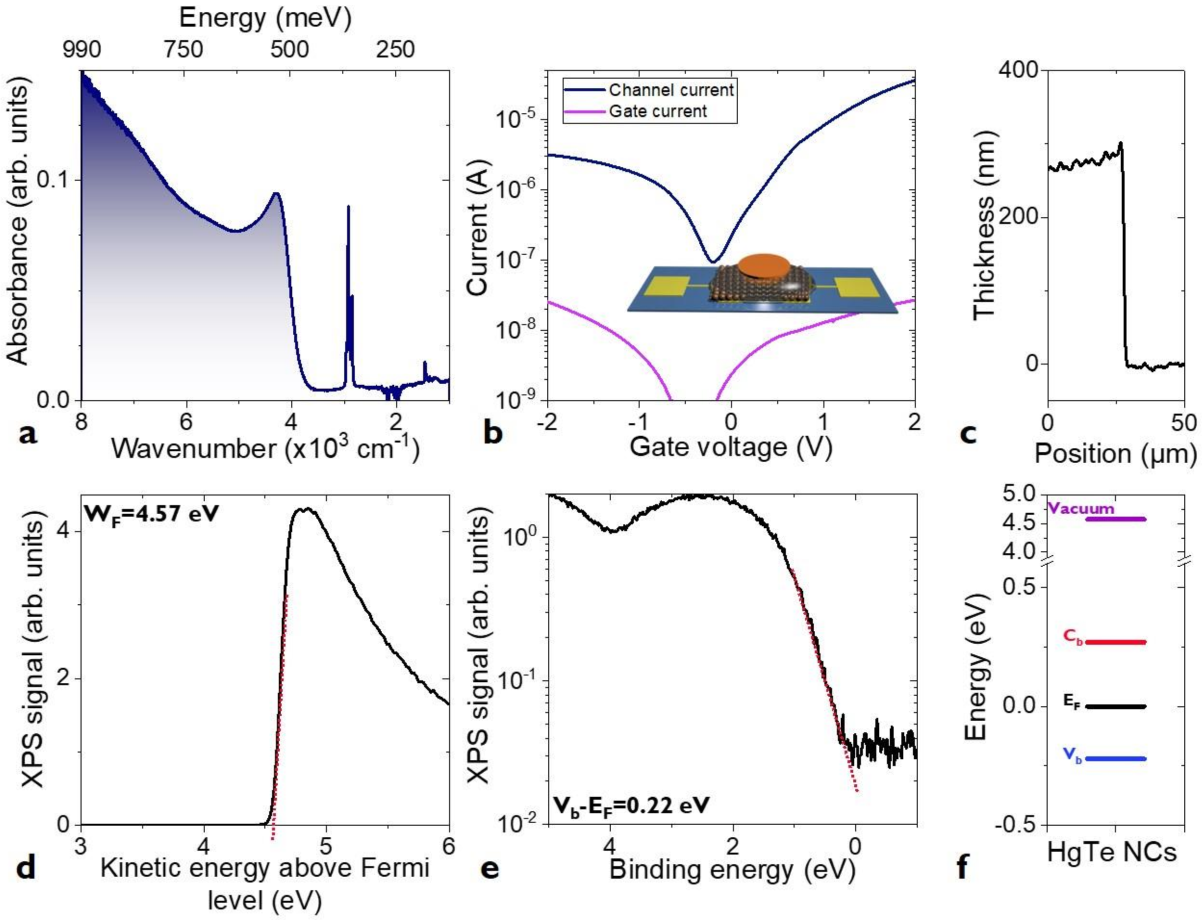

***Supplementary figure 7 : HgTe NC properties**. **a** Absorption spectrum of HgTe nanocrystals. **b** FET curve of a HgTe film on interdigitated electrodes. The gate is an electrolyte ($LiClO_4$ in PEG matrix). A scheme of the FET device is given in inset. **c** Typical thickness profile of an HgTe film used in this study. **d** Cut-off of the secondary electrons as a function of the kinetic energy above the Fermi level for a thin film of HgTe NC film measured by photoemission. **e** Valence band signal as a function of the electron binding energy for a thin film of HgTe NC film. **f** Reconstructed energy diagram of HgTe NCs. The Fermi level is placed at 0 eV (black). The vacuum level (purple) and the valence band (blue) are deduced from photoemission measurements. The conduction band is placed using the band gap deduced from the FTIR spectrum (red).*

Supplementary Note 2: **Mobility of HgTe NC thin film**

We have measured the mobility as a function of temperature for a thin film of HgTe NCs. We observe a drop at low temperature which is responsible for the observed decrease of the responsivity at low temperature, see *Supplementary figure 8*.

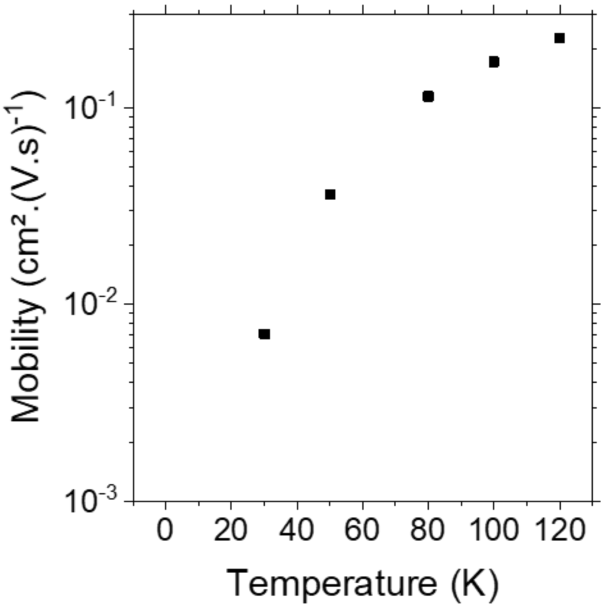

Supplementary figure 8: Mobility as a function of temperature for HgTe NC thin film.

Supplementary Note 3: **HgTe film onto interdigitated 10-µm spaced electrodes**

In order to make a fair comparison of the performance of the nanotrench devices, a film of HgTe on conventional interdigitated electrodes (10 µm spacing) is studied, see *Supplementary figure 9*.

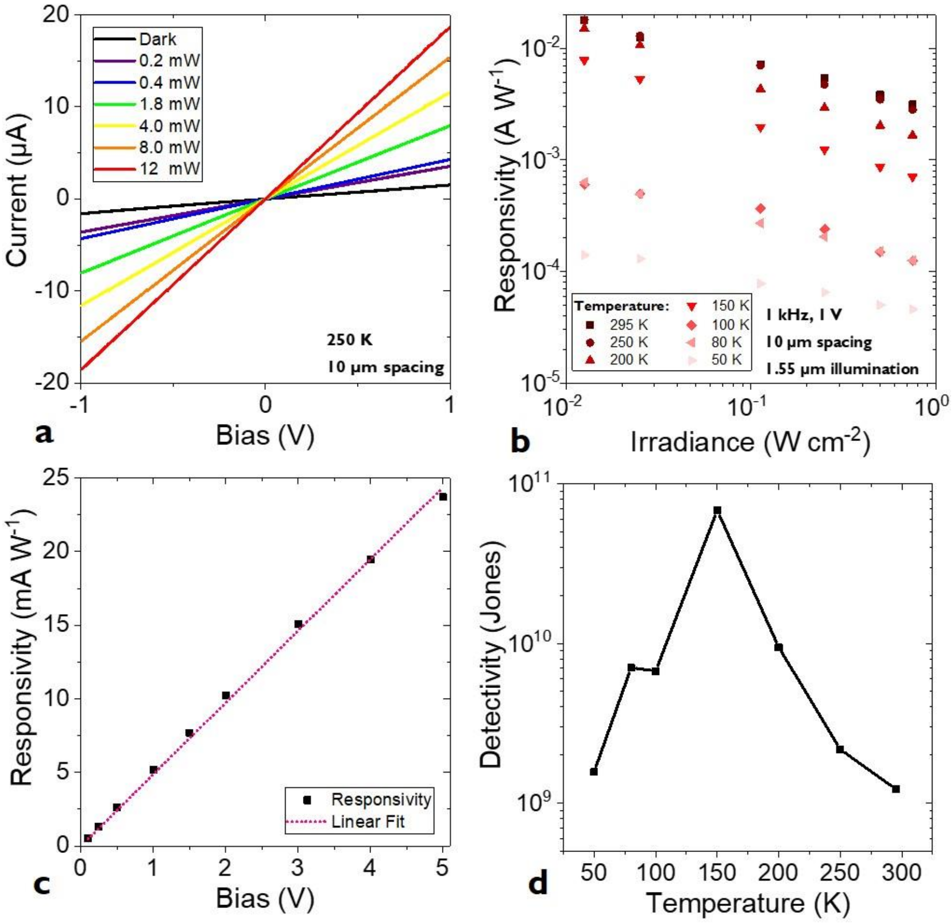

Supplementary figure 9: Study of a HgTe film on interdigitated electrodes. a *I-V curves in dark and under illumination. The light source is a 1.55 µm laser diode at various powers. Temperature is set at 250 K.* ***b*** *Responsivity as a function of the illumination power for various temperatures. The illumination is made with a laser diode at 1.55 µm.* ***c*** *Responsivity as a function of the applied bias.* ***d*** *Specific detectivity as a function of temperature for a HgTe film on interdigitated electrodes.*

Supplementary Note 4: **HgTe film on nanotrench electrodes**

85-nm nanotrench electrodes

A complete study of an HgTe film on 85-nm nanotrench electrodes is conducted, see *Supplementary figure 10*a for a scheme of the device. I-V curves are linear and the conductance increases under illumination indicating clear photoconductive properties, *Supplementary figure 10*b and c. Responsivity reaches few 100 A W^{-1} for both monochromatic (1.55 µm) and blackbody illumination, see *Supplementary figure 10*d and e. This is 4 to 5 orders of magnitude above the performance of HgTe film on interdigitated electrodes. Noise current density still follows a 1/*f* slope, see *Supplementary figure 10*f and the detectivity reaches few 10^{11} Jones with is 3 orders of magnitude higher than conventional electrodes, see *Supplementary figure 10*i and Supplementary figure 9i. Photocurrent spectrum redshifts when the temperature is decreased (*Supplementary figure 10*g) and no Stark effect is visible (*Supplementary figure 10*h).

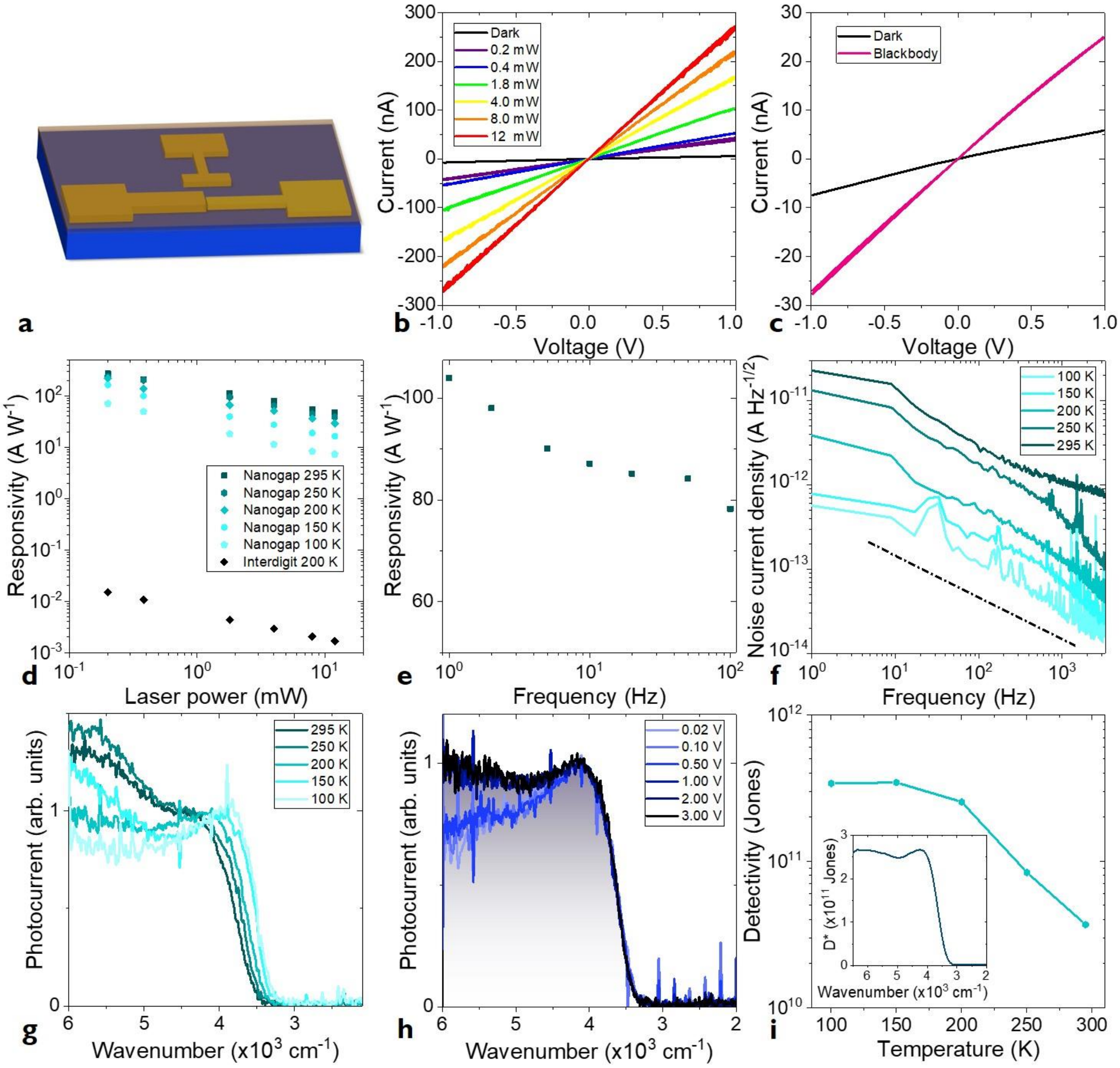

Supplementary figure 10: Study of a HgTe film on 85-nm nanotrench electrodes. a *Scheme of a HgTe NC film on 85-nm nanotrench electrodes.* ***b*** *(resp* ***c****) I-V curves in dark and under illumination. The light source is a 1.55 µm laser diode (resp a black body at 980 °C) at various powers. Temperature is set at 200 K.* ***d*** *Responsivity as a function of illumination power*

for various temperatures. The illumination is made with a laser diode at 1.55 µm. For sake of comparison responsivity for a film on interdigitated electrodes is also shown. ***e*** *Responsivity as a function of frequency at ambient temperature. The illumination is made with a blackbody at 980 °C.* ***f*** *Noise current density as a function of frequency and temperature. The bias is set at 1 V. The dashed line corresponds to the 1/f slope.* ***g*** *Photocurrent spectra at various temperatures of a HgTe film. The bias is set a 1 V.* ***h*** *Normalized photocurrent spectra at various biases. The measurement is made at 200 K.* ***i*** *Detectivity as a function of temperature for a HgTe film on 85-nm-spaced electrodes.*

40-nm nanotrench electrodes

A study of an HgTe film on 40-nm nanotrench electrodes (on LaF_3 substrate) is conducted. A scheme of the device is given *Supplementary figure 11*a. The device with a 40 nm gap has been made on a LaF_3 substrate to be used as an FET. The LaF_3 is an ionic glass where vacancies can be displaced by bias application leading to gate effect. The mobility of the ions at room temperature is high and this create a parasitic parallel resistance to the channel. This is why such device requires cryogenic operations. I-V curves are slightly super-linear but with a clear photoresponse, see Supplementary figure 11b. Responsivity reaches 10^3 A W^{-1} for monochromatic (1.55 µm) illumination, see *Supplementary figure 11*d and the cut-off frequency is above 10 kHz, see *Supplementary figure 11*e. The responsivity follows the transfer curve, see *Supplementary figure 11*c. The noise current density at 200 K is given in Supplementary figure 11f. Its value at 1 kHz is 2.8×10^{-14} A $Hz^{-1/2}$ corresponding to a detectivity of 2×10^{12} Jones.

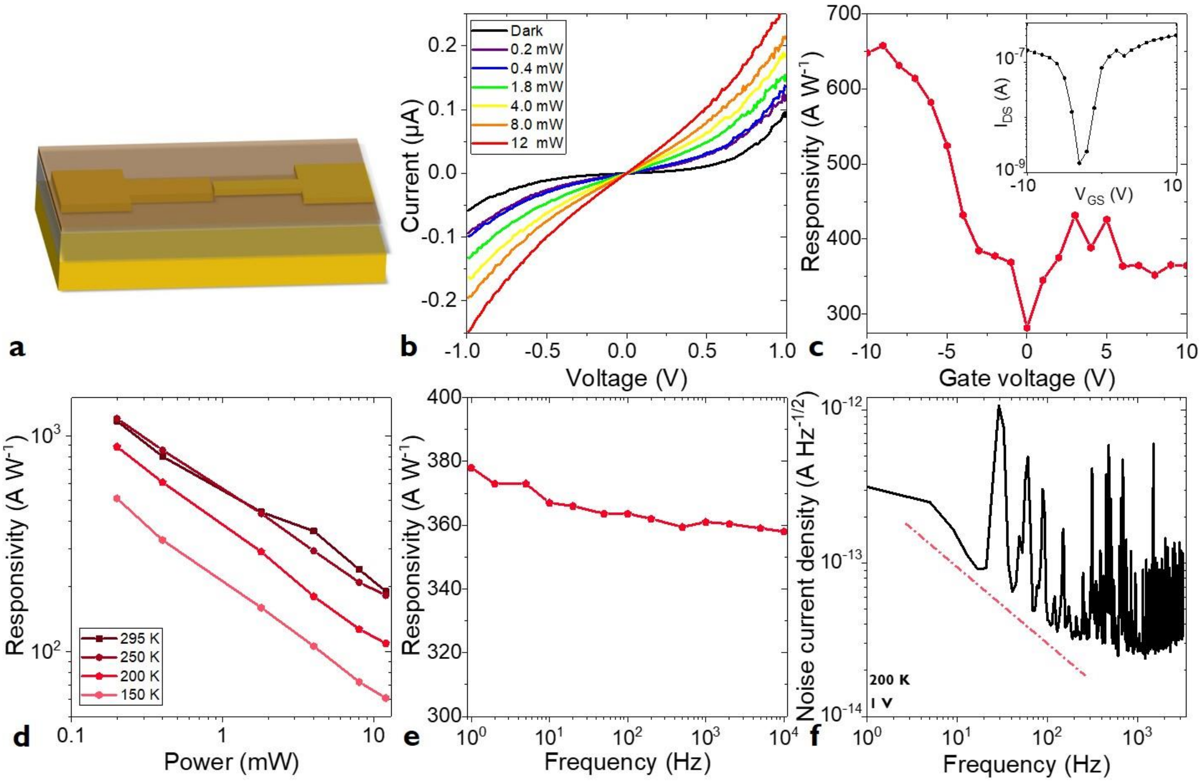

Supplementary figure 11 : Study of a HgTe film on 40-nm nanotrench electrodes. a *Scheme of a HgTe NC film onto 40-nm nanotrench electrodes.* ***b*** *I-V curves in dark and under illumination. The light source is a 1.55 µm laser diode at various powers. Temperature is set at 200 K.* ***c*** *Responsivity as a function of gate voltage. The inset is the dark channel current as a function of gate current.* ***d*** *Responsivity as a function of illumination power for various temperatures. The illumination is made with a laser diode at 1.55 µm.* ***e*** *Responsivity as a*

function of frequency at ambient temperature. The illumination is made with a 1.55 µm laser diode at 4 mW. ***f*** *Noise current density as a function of frequency. The bias is set at 1 V and the temperature at 200 K. The dashed line corresponds to the 1/f slope.*

Supplementary Note 5: **Statistic of nanotrench**

We have measured the dark current and responsivity for a series of nanotech devices which size is around 60 nm, see *Supplementary Figure 12*.

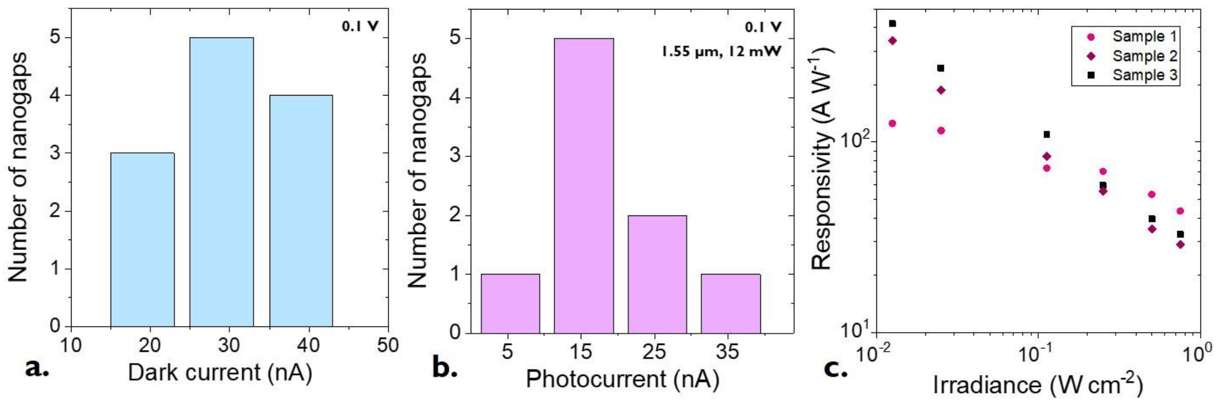

***Supplementary Figure 12: Statistic of nanotrench devices**. Histogram of dark current (**a**) and photocurrent (**b**) for a series of 60-nm nanotrenches at room temperature. **c** Responsivity as a function of the irradiance for three different samples.*

Supplementary Note 6: **Devices with intermediate size**

In order to know the dependency of the detectivity as a function of the size of the interelectrode spacing, several devices with intermediate size are tested.

1.6-µm interelectrode spacing

A study of an HgTe film on 1.6-µm nanotrench electrodes is conducted. I-V curves are linear and the conductance increases under illumination indicating clear photoconductive properties, *Supplementary Figure 13*a. The photocurrent and so the responsivity are independent of the illumination frequency, see *Supplementary Figure 13*b. Responsivity linearly increased by the applied bias, see *Supplementary Figure 13*c. Responsivity achieve 3 A W^{-1}. This is 2 orders of magnitude above the performance of HgTe film on interdigitated electrodes. Noise current density still follows a 1/*f* slope (until the limit of the setup is achieved), see *Supplementary Figure 13e* and the detectivity reaches 10^{11} Jones at 150 K, see *Supplementary Figure 13*f.

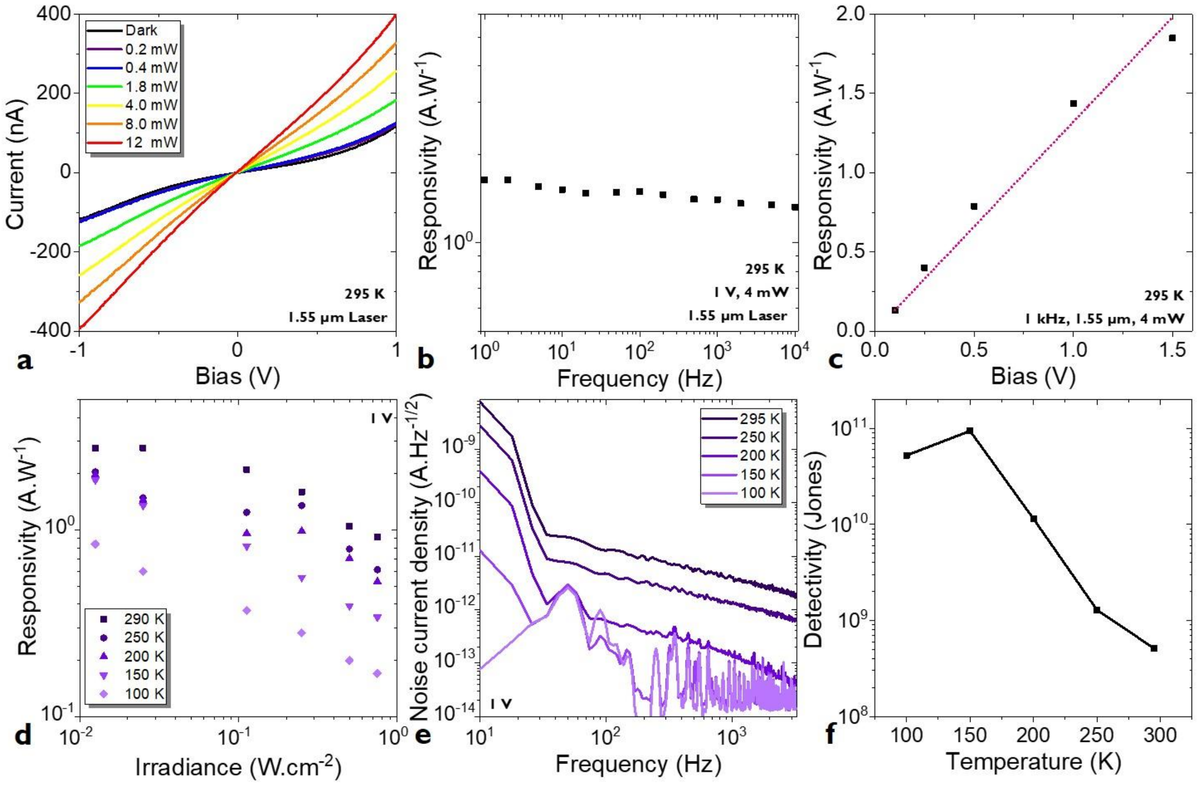

***Supplementary Figure 13: Study of a film on electrodes with a 1.6-µm spacing. a** I-V curves in dark and under illumination. The light source is a 1.55 µm laser diode at various powers. Temperature is set at 295 K. **b** Responsivity as a function of frequency at ambient temperature. The illumination is made with a 1.55-µm laser diode at 4 mW. **c** Responsivity as a function of the applied bias. The illumination is made with a 1.55-µm laser diode at 4 mW and chopped at 1 kHz. **d** Responsivity as a function of the illumination power for various temperatures. The illumination is made with a laser diode at 1.55 µm and chopped at 1 kHz. **e** Noise current density as a function of frequency and temperature. The bias is set at 1 V. **f** Detectivity at 1 kHz as a function of temperature for a HgTe film on 1.6-µm-spaced electrodes. Illumination is made with a 1.55-µm laser diode at 0.2 mW.*

730-nm interelectrode spacing

A study of an HgTe film on 730-nm nanotrench electrodes is conducted. I-V curves are sur-linear and the conductance increases under illumination indicating clear photoconductive properties, *Supplementary Figure 14*a. Responsivity achieve 3 A W^{-1}. This is 2 orders of magnitude above the performance of HgTe film on interdigitated electrodes. Noise current density still follows a 1/*f* slope (until the limit of the setup is achieved), see *Supplementary Figure 14c* and the detectivity reaches 5x10^{10} Jones at 150 K, see *Supplementary Figure 14*d.

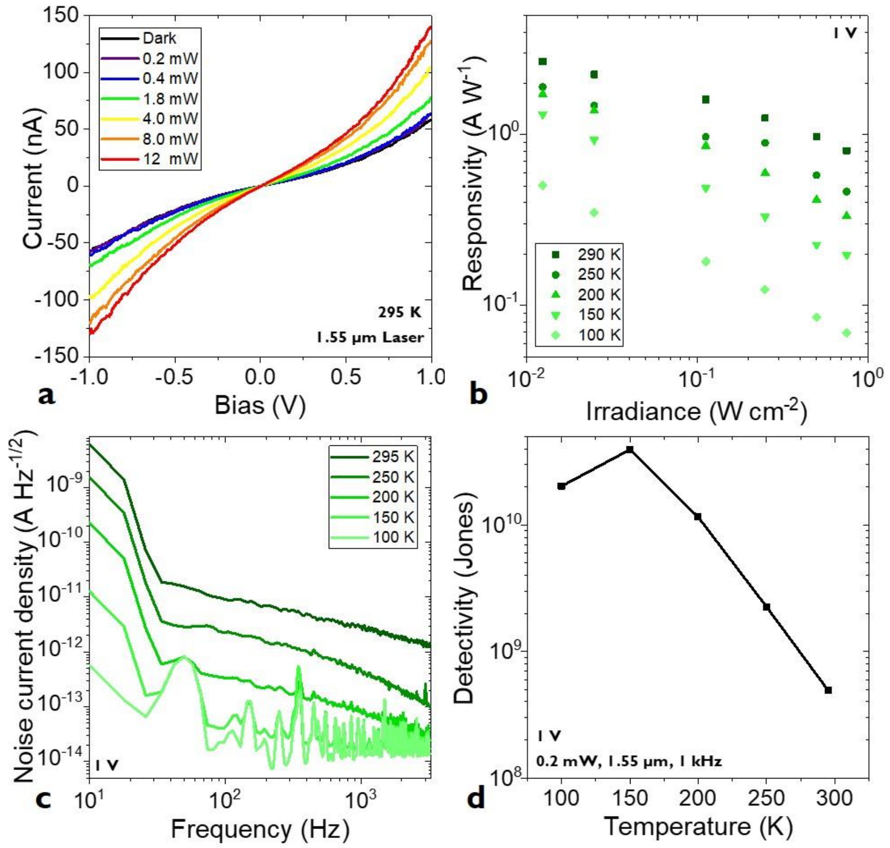

Supplementary Figure 14: Study of a film on electrodes with a 730-nm spacing. a *I-V curves in dark and under illumination. The light source is a 1.55 µm laser diode at various powers. Temperature is set at 295 K.* ***b*** *Responsivity as a function of the illumination power for various temperatures. The illumination is made with a laser diode at 1.55 µm and chopped at 1 kHz.* ***c*** *Noise current density as a function of frequency and temperature. The bias is set at 1 V.* ***d*** *Detectivity at 1 kHz as a function of temperature for a HgTe film on 730-nm-spaced electrodes. Illumination is made with a 1.55-µm laser diode at 0.2 mW.*

250-nm interelectrode spacing

A study of an HgTe film on 240-nm nanotrench electrodes is conducted. I-V curves are sur-linear and the conductance increases under illumination indicating clear photoconductive properties, *Supplementary Figure 15*a. Responsivity achieve 11 A W^{-1}. This is 3 orders of magnitude above the performance of HgTe film on interdigitated electrodes. Noise current density still follows a 1/*f* slope, see *Supplementary Figure 15*c and the detectivity reaches $2x10^{10}$ Jones at 100 K, see *Supplementary Figure 15*d.

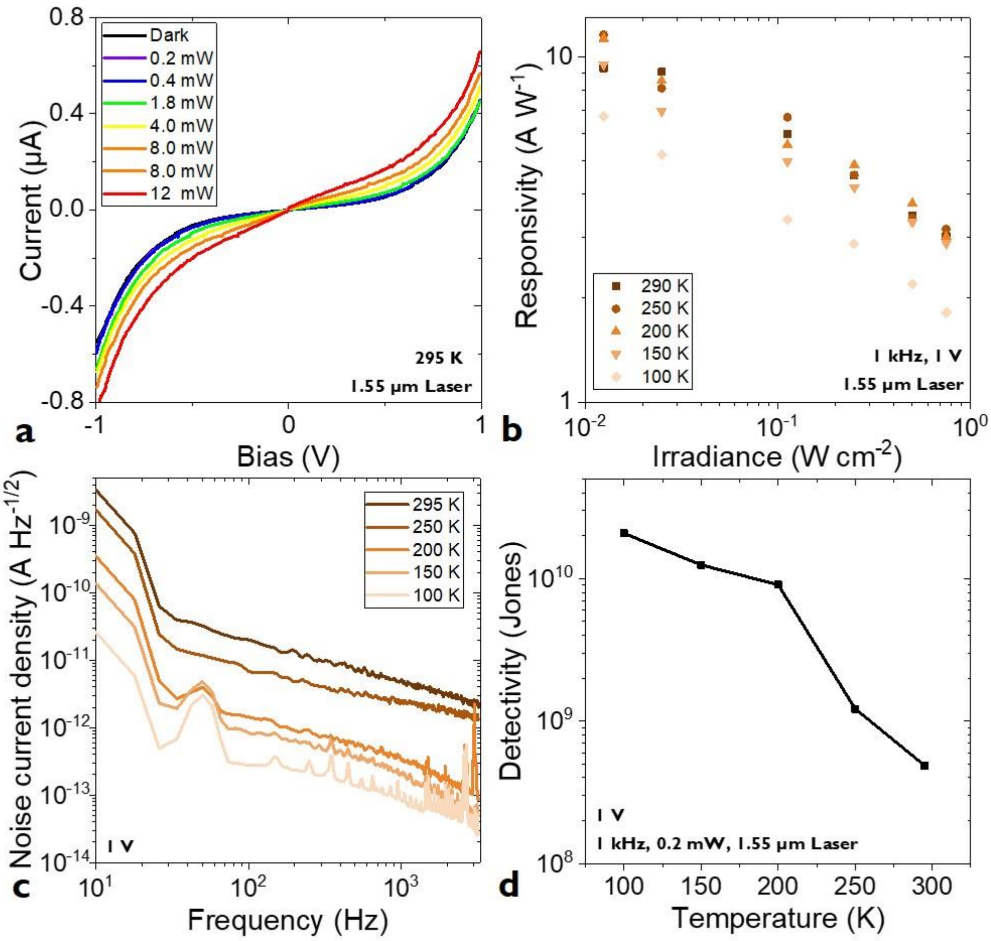

Supplementary Figure 15: Study of a film on electrodes with a 240-nm spacing. a *I-V curves in dark and under illumination. The light source is a 1.55 µm laser diode at various powers. Temperature is set at 295 K.* ***b*** *Responsivity as a function of the illumination power for various temperatures. The illumination is made with a laser diode at 1.55 µm and chopped at 1 kHz.* ***c*** *Noise current density as a function of frequency and temperature. The bias is set at 1 V.* ***d*** *Detectivity at 1 kHz as a function of temperature for a HgTe film on 240-nm-spaced electrodes. Illumination is made with a 1.55-µm laser diode at 0.2 mW.*

Responsivity for different sizes

The responsivity measured for all devices are summarized in *Supplementary Figure 16*. A trend is clearly visible: the smaller the spacing the higher the responsivity.

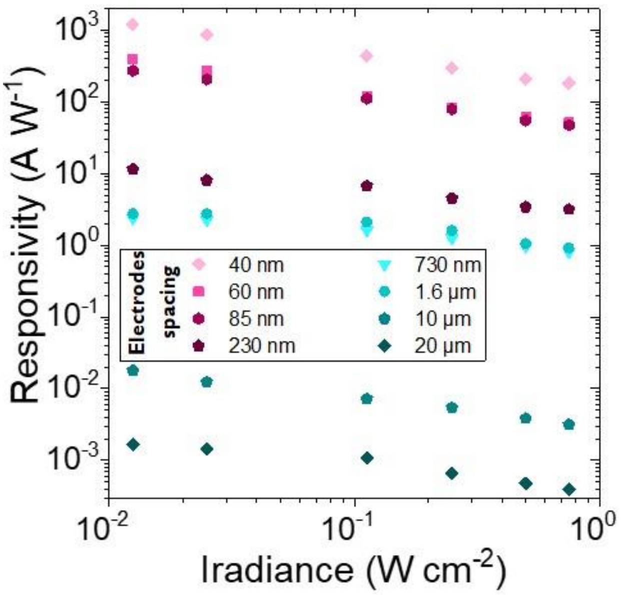

Supplementary Figure 16: Responsivity of the device for different interelectrode spacing.

Supplementary Note 7: **Linearity of the response**

The responsivity appears to be linear for all types of devices, see *Supplementary Figure 17*.

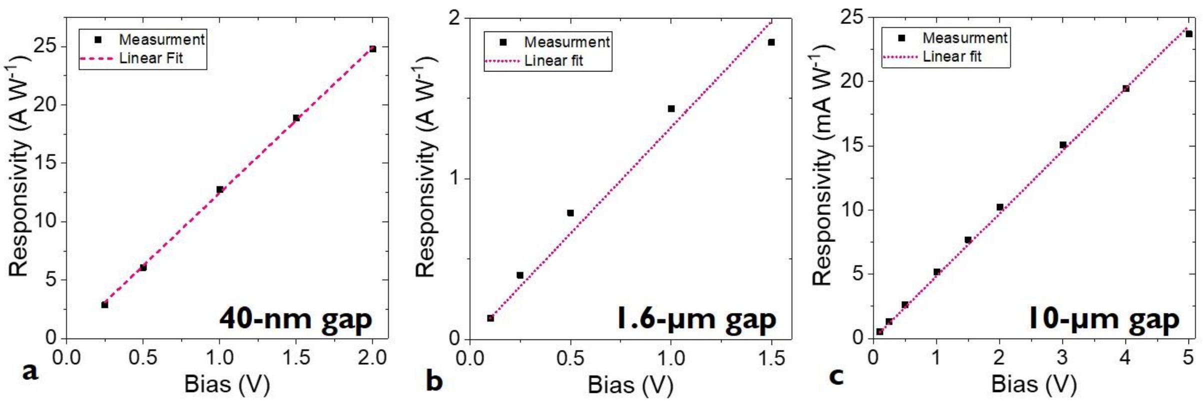

Supplementary Figure 17: Linearity of the photoresponse*. Responsivity as a function of the applied bias for 3 sizes of trench 40 nm (**a**), 1.6 µm (**b**) and 10 µm (**c**). The dots are experimental data, while the pink dashed lines correspond to a linear fit.*

Supplementary Note 7: **Electromagnetic simulations**

Inputs for the simulation

Electromagnetic simulations are achieved with Matlab Reticolo library based on Rigorous Coupled-Wave Analysis[3] (RCWA). Necessary inputs for this simulation are (i) the design of the structure and (ii) the optical index of each layer composing the design. The layers of the design are given in *Supplementary table 1*. This simulation using periodic boundary condition, the period is set at 10 µm in order to reduce the interaction of a nanotrench with its neighbors. This design model for the simulation does not take into account the asymmetry (*i.e.* the thickness difference, neither possible rounding due to tilted evaporation) of both nanoelectrodes due to the tilted evaporation.

Layer	HgTe on a Au mirror	HgTe on a Nanogap electrodes
**1**	External environment: Air	External environment: Air
**2**	HgTe NC film: 300 nm	HgTe NC film: 300 nm
**3**	Gold mirror: 60 nm	Gold with a 40-nm nanogap filed with HgTe: 60 nm
**4**	External environment: Si	External environment: Si

Supplementary table 1 : Input layer of the electromagnetic simulations.

Regarding the optical indices, n_{air}=1 is taken. For gold and silicon, the indices are taken from ref [4] and [5]. For the nanocrystals, the real part of the optical index is taken equal to 2. This value is commonly used NC thin films. For the imaginary part, we use the absorption shape of the NCs and we normalized its value at the exciton peak. To do so we measure the absorption coefficient to be $3x10^3$ cm^{-1} which give k=0.04 at the band edge.

A second set of simulation is done with COMSOL in order to study the absorption within the different parts of the device. Calculations have been achieved using COMSOL Multiphysics software with RF Module and Electromagnetic Waves, Frequency Domain physics. Input port has been set with user defined plane wave-front. Domain is surrounded by perfectly matched layers (PML). The mesh is made of triangular elements whose maximum size has been refined is the nanotrench area and is set to 1 nm. In QD layer, maximum element size is 100 nm. These values are more than sufficient to properly describe waveguide propagation and peak effects.

Simulation results

The first results given by those simulations are the absorption spectra of both designs, see *Supplementary figure 18*. Sharp peaks (with small magnitude) in the nanotrench spectrum are numerical artefacts. For a same film thickness, both spectra have a similar shape. This result confirms that the nanotrench does not induce a large resonance. More, for a 300 nm film, the total absorption of the device is around 25%.

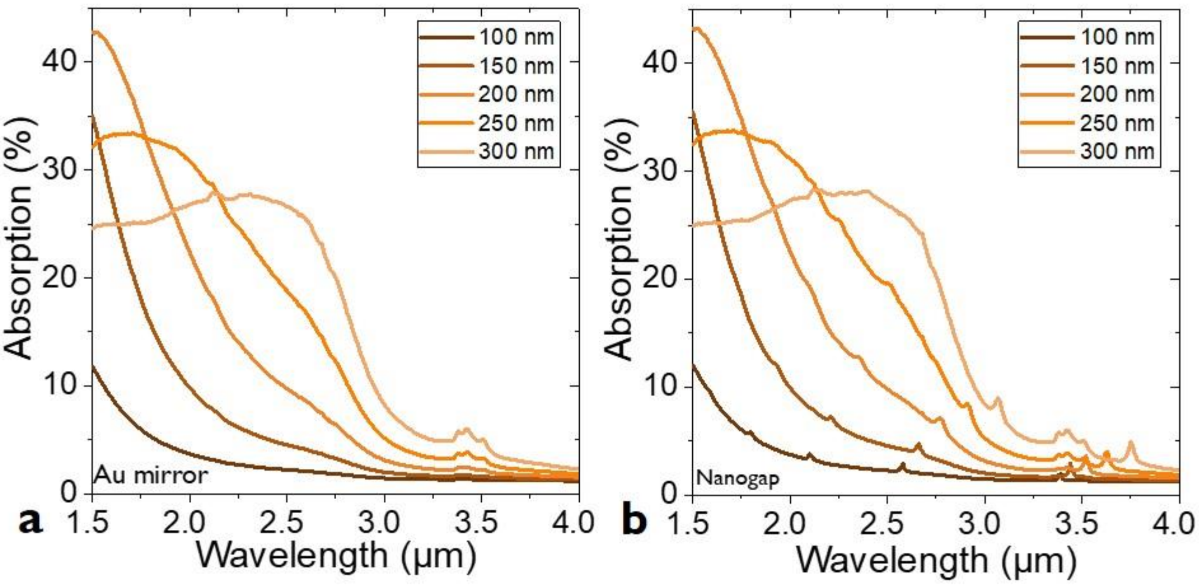

***Supplementary figure 18: Simulated absorption spectra from electromagnetic simulations. a** (resp **b**) Simulated absorption spectra of HgTe NC films on a gold mirror (resp on nanotrench electrodes) for various HgTe film thicknesses.*

To complete the understanding of the absorption within the nanotrench device we used electromagnetic simulations using COMSOL to calculate the spectra within the nanotrench, the electrodes and the top QD film for both polarizations, see *Supplementary figure 19*. First there is no difference between transverse electric (TE) and transverse magnetic ™ polarizations for absorption in the top film and the metallic contacts. While there are few orders of magnitude difference between TE and TM within the nanotrench. Secondly, in TM polarization we see that the absorption within the nanotrench represents only 1 percent of the total QD absorption. This further confirm that the active area is indeed the nanotrench.

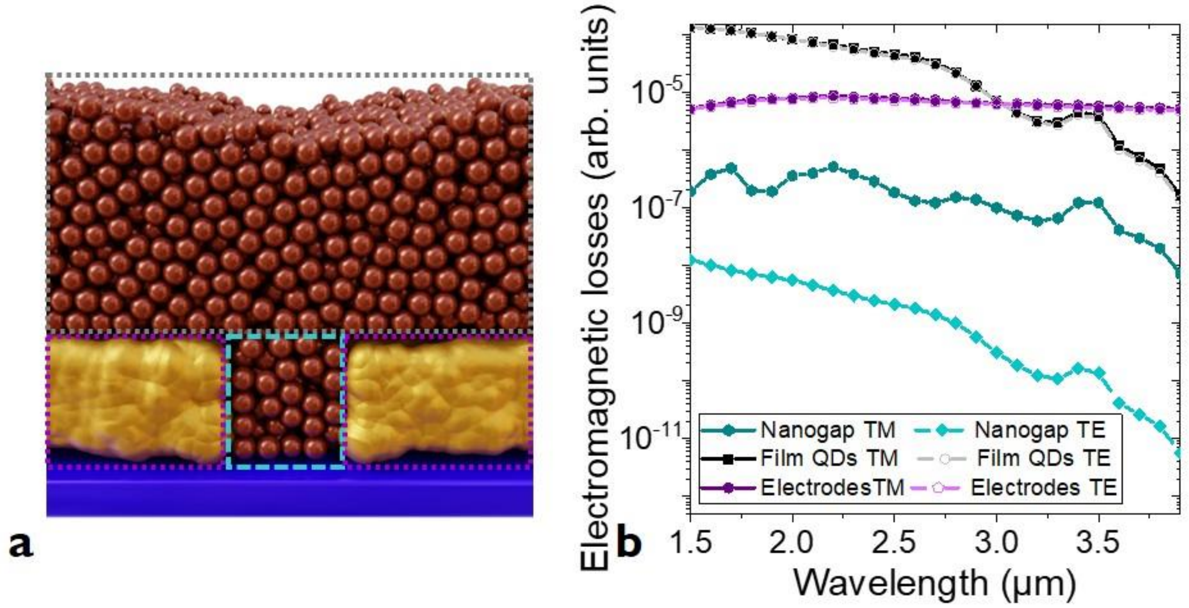

***Supplementary figure 19: Simulated absorption spectra from electromagnetic simulation. a** Scheme of the nanotrench and definitions of the parts involved in electromagnetic losses: the nanotrench (blue), the metallic contacts (pink) and the top film*

(gray). ***b*** *Simulated absorption spectra of a 300-nm HgTe NC film on a 80-nm nanotrench for different parts of the device and under different polarizations.*

In *Supplementary figure 20*, field maps of both structures (calculated with RCWA method) are given. As expected, no polarization dependence is observed for a NC film on the gold mirror, see *Supplementary figure 20*a-c. No evidence of resonances appears. For nanotrench electrodes, due to the break of symmetry, the field depends on the polarization, see *Supplementary figure 20*d-i. In particular, a clear enhancement of the x-component of the electric field in TM mode appears in the nanotrench, see *Supplementary figure 20*e. This enhancement leads to an increase of the absorption of a factor about 10 in the TM polarization.

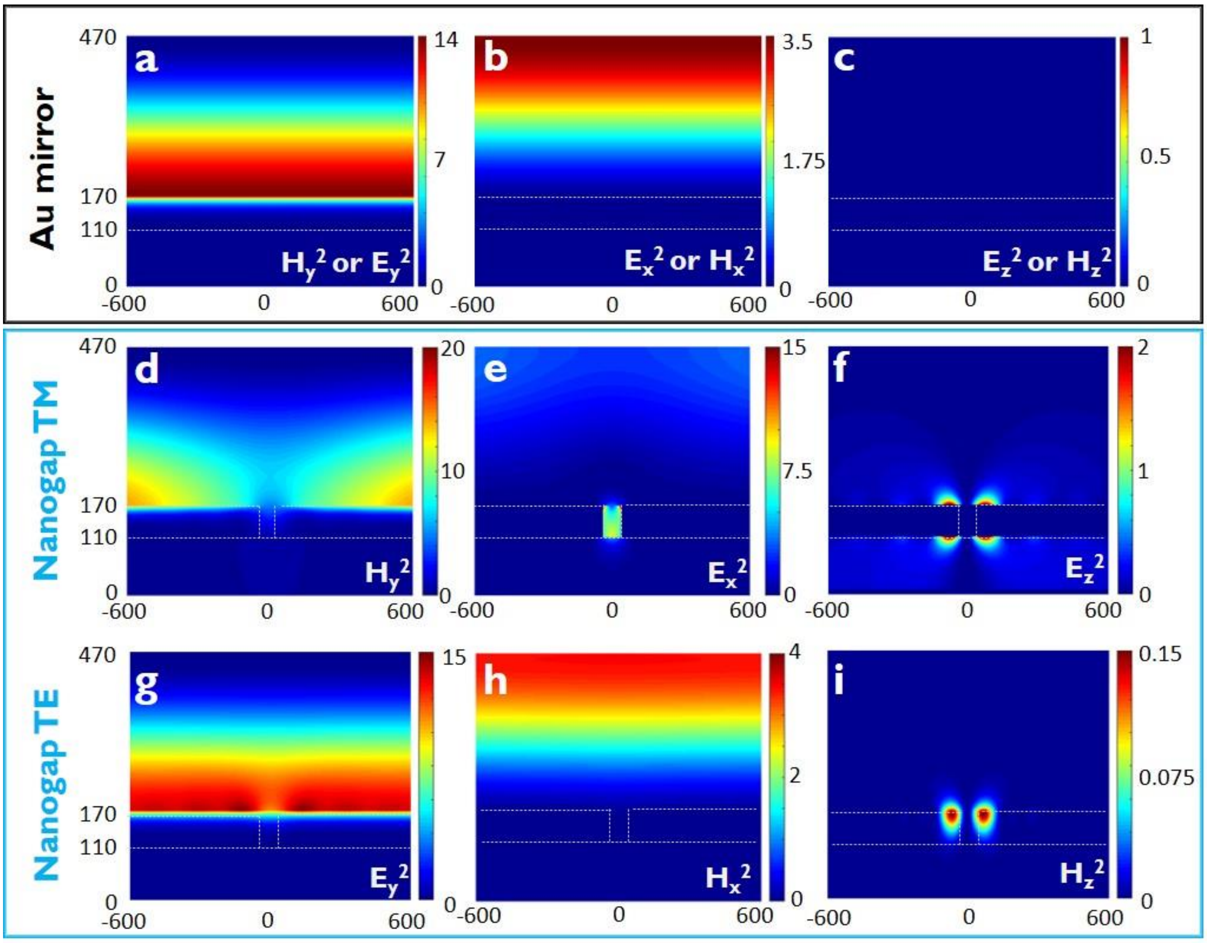

Supplementary figure 20: Simulated field map. a *Simulated field map of the y component of the magnetic (or electric) field in TM (or TE) polarization for a film of HgTe on a Au mirror.* ***b*** *Simulated field map of the x component of the electric (or magnetic) field in TM (or TE) polarization for a film of HgTe on a Au mirror.* ***c*** *Simulated field map of the z component of the electric (or magnetic) field in TM (or TE) polarization for a film of HgTe on a Au mirror.* ***d*** *(resp* ***g****) Simulated field map of the y component of the magnetic (resp electric) field in TM (resp TE) polarization for a film of HgTe on nanotrench electrodes.* ***e*** *(resp* ***h****) Simulated field map of the x component of the electric (resp magnetic) field in TM (or TE) polarization for a film of HgTe on nanotrench electrodes.* ***f*** *(resp* ***i****) Simulated field map of the z component of the electric (or magnetic) field in TM (or TE) polarization for a film of HgTe on nanotrench electrodes. The axes are in nm. The x-axis corresponds to the x direction in simulation. The y-axis corresponds to the device thickness and to the z direction in simulation. The scalebar represents the enhancement of each field.*

Supplementary Note 8: **Tight binding simulations**

The calculation of the overlap between electron and hole wavefunctions in 3D is made using tight binding model described in ref [6]. The overlap is usually calculated as the inner product between the electron (φ_e) and hole (φ_h) wavefunctions. The problem here is that $\langle\varphi_e|\varphi_\mathrm{h}\rangle = 0$ because the two solutions of the same Hamiltonian are orthogonal. This orthogonality comes from the Bloch part of the wavefunctions, and not from the envelope that is the interesting part in our case.

To overcome this problem, the calculation is made according to the following procedure. First the weight of the wavefunction on each atom is calculated according to these formulas:

$$c_i \equiv \int_{\Omega_i} \left|\varphi_e(r)\right|^2 dV$$

$$v_i \equiv \int_{\Omega_i} \left|\varphi_\mathrm{h}(r)\right|^2 dV$$

where Ω_i is the volume occupied by the atom i.

Secondly the overlap is defined as:

$$S = \frac{2\sum_i c_i v_i}{\sum_i |c_i|^2 + \sum_i |v_i|^2}$$

Using this definition, S is equal to one when the two wavefunctions are identical and goes to zero when those two do not overlap.

Supplementary references

1. Dayen, J.-F., Faramarzi, V., Pauly, M., Kemp, N. T., Barbero, M., Pichon, B. P., Majjad, H., Begin-Colin, S. & Doudin, B. Nanotrench for nano and microparticle electrical interconnects. *Nanotechnology* **21**, 335303 (2010).
2. Jagtap, A., Martinez, B., Goubet, N., Chu, A., Livache, C., Gréboval, C., Ramade, J., Amelot, D., Trousset, P., Triboulin, A., Ithurria, S., Silly, M. G., Dubertret, B. & Lhuillier, E. Design of a Unipolar Barrier for a Nanocrystal-Based Short-Wave Infrared Photodiode. *ACS Photonics* **5**, 4569–4576 (2018).
3. Hugonin, J.-P. & Lalanne, P. Reticolo Software for Grating Analysis. doi: 10.5281/zenodo.4419063 (2005).
4. Olmon, R. L., Slovick, B., Johnson, T. W., Shelton, D., Oh, S.-H., Boreman, G. D. & Raschke, M. B. Optical dielectric function of gold. *Phys. Rev. B* **86**, 235147 (2012).
5. Chandler-Horowitz, D. & Amirtharaj, P. M. High-accuracy, midinfrared (450cm−1⩽ω⩽4000cm−1) refractive index values of silicon. *J. Appl. Phys.* **97**, 123526 (2005).
6. Allan, G. & Delerue, C. Tight-binding calculations of the optical properties of HgTe nanocrystals. *Phys. Rev. B* **86**, 165437 (2012).
